# 1α,25(OH)_2_-3-Epi-Vitamin D_3_, a Natural Physiological Metabolite of Vitamin D_3_: Its Synthesis, Biological Activity and Crystal Structure with Its Receptor

**DOI:** 10.1371/journal.pone.0018124

**Published:** 2011-03-31

**Authors:** Ferdinand Molnár, Rita Sigüeiro, Yoshiteru Sato, Clarisse Araujo, Inge Schuster, Pierre Antony, Jean Peluso, Christian Muller, Antonio Mouriño, Dino Moras, Natacha Rochel

**Affiliations:** 1 Institut de Génétique et de Biologie Moléculaire et Cellulaire (IGBMC), Institut National de Santé et de Recherche Médicale (INSERM) U964/Centre National de Recherche Scientifique (CNRS) UMR 7104/Université de Strasbourg, Illkirch, France; 2 School of Pharmacy, Faculty of Health Sciences, University of Eastern Finland, Kuopio, Finland; 3 Departamento de Quimica Organica, Universidad de Santiago de Compostela and Unidad Asociada al CSIC, Santiago de Compostela, Spain; 4 Institute of Pharmaceutical Chemistry, University of Vienna, Vienna, Austria; 5 Faculty of Pharmacy, Institut Gilbert Laustriat, UMR 7175 CNRS, University of Strasbourg, Illkirch, France; Roswell Park Cancer Institute, United States of America

## Abstract

**Background:**

The 1α,25-dihydroxy-3-epi-vitamin-D_3_ (1α,25(OH)_2_-3-epi-D_3_), a natural metabolite of the seco-steroid vitamin D_3_, exerts its biological activity through binding to its cognate vitamin D nuclear receptor (VDR), a ligand dependent transcription regulator. *In vivo* action of 1α,25(OH)_2_-3-epi-D_3_ is tissue-specific and exhibits lowest calcemic effect compared to that induced by 1α,25(OH)_2_D_3_. To further unveil the structural mechanism and structure-activity relationships of 1α,25(OH)_2_-3-epi-D_3_ and its receptor complex, we characterized some of its *in vitro* biological properties and solved its crystal structure complexed with human VDR ligand-binding domain (LBD).

**Methodology/Principal Findings:**

In the present study, we report the more effective synthesis with fewer steps that provides higher yield of the 3-epimer of the 1α,25(OH)_2_D_3_. We solved the crystal structure of its complex with the human VDR-LBD and found that this natural metabolite displays specific adaptation of the ligand-binding pocket, as the 3-epimer maintains the number of hydrogen bonds by an alternative water-mediated interaction to compensate the abolished interaction with Ser278. In addition, the biological activity of the 1α,25(OH)_2_-3-epi-D_3_ in primary human keratinocytes and biochemical properties are comparable to 1α,25(OH)_2_D_3_.

**Conclusions/Significance:**

The physiological role of this pathway as the specific biological action of the 3-epimer remains unclear. However, its high metabolic stability together with its significant biologic activity makes this natural metabolite an interesting ligand for clinical applications. Our new findings contribute to a better understanding at molecular level how natural metabolites of 1α,25(OH)_2_D_3_ lead to significant activity in biological systems and we conclude that the C3-epimerization pathway produces an active metabolite with similar biochemical and biological properties to those of the 1α,25(OH)_2_D_3_.

## Introduction

The 1α,25-dihydroxyvitamin D_3_ (1α,25(OH)_2_D_3_ or calcitriol), is the most active form of vitamin D_3_ and mediates its pleiotropic effects through VDR activation, which heterodimerizes with retinoid X receptor (RXR). VDR-induced genomic action results in growth inhibition of lymphomas, breast or prostate primary tumor cells, renal osteodystrophy, osteoporosis, psoriasis or autoimmune diseases [1], [2]. Consequently, VDR is an exquisite therapeutic target to combat human metabolic diseases and uncontrolled cell proliferation in many tissues [3]–[5]. In addition 1α,25(OH)_2_D_3_ is a key regulator of calcium and phosphate homeostasis and bone metabolism but its intrinsic hypercalcemic effect prevents its use in therapeutical applications [6].

1α,25(OH)_2_D_3_ is subjected to enzymatic inactivation via two major pathways leading to C-24 and C-23 hydroxylated metabolites in various tissues [7]–[17]. While the side chain oxidation is a general pathway associated to inactivation, another metabolite modified at the A-ring, the 1α,25(OH)_2_-3-epi-D_3_, has been shown to retain significant biological activity compared to the natural hormone [18], [19]. The 1α,25(OH)_2_-3-epi-D_3_ was initially identified in the culture of human neonatal keratinocytes [20], [21]. Further *in vivo* studies have characterized the occurrence of a C-3 epimerization pathway [22]. Indeed, this natural vitamin D_3_ metabolite was detected in serum of rats treated with pharmacological doses of 1α,25(OH)_2_D_3_, and may therefore play an important physiological role by buffering the level of 1α,25(OH)_2_D_3_. In addition, significant accumulation of 1α,25(OH)_2_-3-epi-D_3_ was observed in different human adenocarcinoma cell lines such as colon-derived Caco-2 cells[23] or NCI-H441 pulmonary cells [24]. Moreover, 1α,25(OH)_2_-3-epi-D_3_ was readily quantified in bovine parathyroid cells, [25] rat osteoblastic UMR 106 and Ros17/2.8 cells [26].

**Figure 1:**
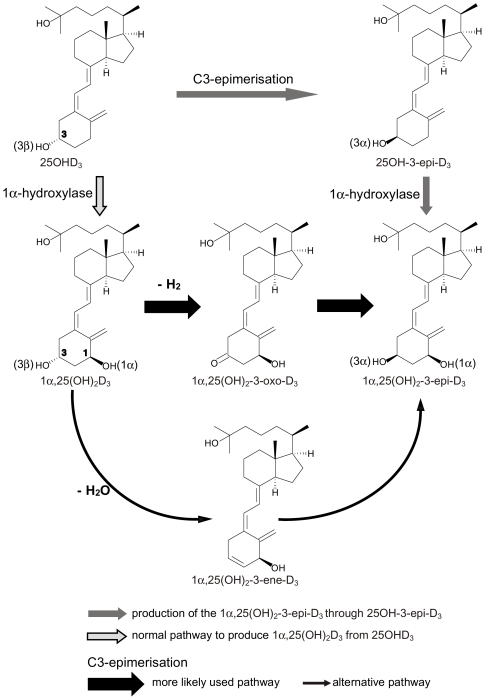
Proposed pathway of the 1α,25(OH)_2_-3-epi-D_3_ production [18]. The reaction is initiated via A-ring C3-epimerization, where the C-3 hydroxyl moiety is changed from β to its diastereomer α. Two distinct pathways may be employed by cells to generate 1α,25(OH)_2_-3-epi-D_3_. The first, more likely used pathway, starts with dehydrogenation catalyzed by yet unidentified enzyme leading to a keto-intermediate, which is converted most probably by the same enzyme to the final product 1α,25(OH)_2_-3-epi-D_3_. The second one uses dehydration and a subsequent hydroxylation at C-3 α position.

The production of 1α,25(OH)_2_-3-epi-D_3_ is initiated via A-ring C3-epimerization (Figure 1), where the C-3 hydroxyl moiety is changed from position β to its diastereomer α. The enzymes responsible for the C3-epimerization have not been identified to present date. It was also proposed by Reddy *et al*. that this pathway might be used for metabolites that resist inactivation through C-24 oxidation [18] a phenomenon well characterized in the bile acid metabolism where the reaction is catalyzed by bile acid hydroxysteroid dehydrogenase [27]. This pathway plays also a major role in the activation and/or inactivation of steroid hormones such as androgens [28].

Despite a lower binding affinity than calcitriol, 1α,25(OH)_2_-3-epi-D_3_ possess significant biological activity only in specific tissues where it is produced [29]. The transcriptional response of the 1α,25(OH)_2_-3-epi-D_3_ compound varies for different VDR-regulated genes in different tissues. For instance, it shows lower activation of osteocalcin gene and lower HL60 differentiation [30] but has almost equipotent activity to 1α,25(OH)_2_D_3_ in inhibiting cellular proliferation in keratinocytes [19] and in suppressing parathyroid secretion in bovine parathyroid cells [25]. These *in vitro* properties associated with its low calcemic activity [31], [32] assign potential therapeutic interest to this compound.

To further unveil, the structural mechanism and structure-activity relationships of 1α,25(OH)_2_-3-epi-D_3_/hVDR-LBD complex, we describe a more effective synthetic route to the synthesis of 1α,25(OH)_2_-3-epi-D_3_, some of its *in vitro* biological properties and the crystal structure of its complex with hVDR LBD.

## Results and Discussion

### Synthesis of the 1α,25(OH)_2_-3-epi-D_3_

The synthesis of the target 1α,25(OH)_2_-3-epi-D_3_ (**1**, Scheme S1) was first described by Okamura's group at Riverside from (*R*)-carvone using the dienyne approach (13 steps, 8.5%) [33]. We describe here an efficient and alternative convergent synthesis of **1** from (*S*)-carvone (9 steps, 13%) that features a palladium catalyzed tandem process that produces the vitamin D triene unit stereoselectively in one pot by coupling enol triflate **3** (A-ring fragment) with an alkenyl metal intermediate **2** (CD-side chain fragment) [34]. For reproducibility reasons we employed Indium intermediates (M = InR_2_) instead of Zinc intermediates [35]–[37].

### Synthesis of the A-ring fragment 3

Our synthesis starts with commercial (*S*)-carvone (**4**, Scheme S2), which was reduced under Luche conditions [38] to alcohol **5a** and its epimer **5b** (9∶1 ratio as determined by ^1^H-NMR). The mixture of alcohols **5** was subjected to Sharpless epoxidation [39] to provide the desired epoxyalcohol **6a** (58% yield, two steps) and the starting ketone **4** (28%). The formation of **4** can be explained by oxidation of **5a** through the corresponding chair-like equatorially oriented vanadium ester intermediate. *Tert*-butyldimethylsilyl protection of **6a** gave **6b** in 96% yield. Side-chain degradation on **6b** by Daniewski's method [40] afforded alcohol **7a** (71%), which was protected to **7b** in the usual way (91%). Epoxide **7b** was converted in 77% yield to dibromide **8b** by the two-step sequence: 1) oxidative cleavage with periodic acid; 2) Corey-Fuchs side-chain extension [41]. Finally, consecutive treatment of **8b** with lithium diisopropylamide and *n*-butyllithium followed by trapping of the resulting enolate with *N*-(5-Cl-2-pyridil)bis(triflate) gave the desired enol triflate **3** in 76% yield [42].

### Synthesis of the upper fragment 2 and 1α,25(OH)_2_-3-epi-D_3_ (1b)

Alkenyl bromide **10** was prepared from ketone **9** by a modified [43] Trost procedure [44]. Treatment of a mixture of bromide **10** and indium trichloride with *tert*-butyllithium, and coupling of the resulting indium intermediate **2a** with enol triflate **3** in the presence of catalytic amounts of (Ph_3_P)_4_Pd and (dppf)PdCl_2_, gave, after desilylation, the desired metabolite **1b** in 58% yield (Scheme S3). The detailed synthesis is described in the Methods S1.

### 1α,25(OH)_2_D_3_ and 1α,25(OH)_2_-3-epi-D_3_ show similar properties in coactivator peptide recruitment

**Figure 2:**
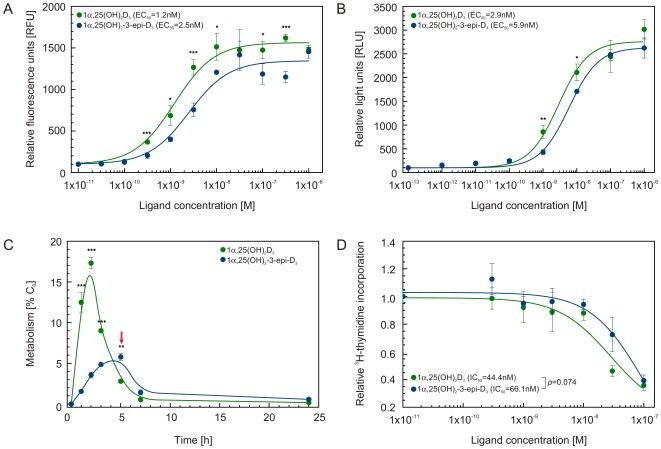
1α,25(OH)_2_D_3_ and 1α,25(OH)_2_-3-epi-D_3_ show similar biological properties. (A) Coactivator peptide recruitment assay was performed using AlphaScreen method in the presence of increasing concentrations of either 1α,25(OH)_2_D_3_ (green circles) or 1α,25(OH)_2_-3-epi-D_3_ (blue circles). The data represents two independent measurements in triplicates for which the mean and the S.D. of the mean was calculated. (B) Transactivation assays were performed in human breast cancer cells MCF7 cells with subsequent treatments of the increasing concentrations of either 1α,25(OH)_2_D_3_ (green circles) or 1α,25(OH)_2_-3-epi-D_3_ (blue circles). For every triplicate the mean and the S.D. were calculated. (C) Metabolism of ^3^H-25(OH)D_3_ in human keratinocytes. Kinetics of the primary metabolite 1α,25(OH)_2_D_3_ and its 3-epimer, is shown. The time point 5 h, where the 1α,25(OH)_2_-3-epi-D_3_ is the major metabolite is highlighted with red arrow. Confluent keratinocytes derived from lid skin were incubated in KGM (0.06 mM calcium) with 20.6 nM ^3^H[26], [27]-25(OH)D_3_ for the indicated time periods. CHCl_3_-extracts of the incubations were analyzed on Zorbax-Sil and individual metabolites identified by matching with authentic reference compounds and quantified as described in Materials and Methods. Data (± SD) was calculated from duplicate experiment. (D) Anti-proliferative cellular effect of 1α,25(OH)_2_D_3_ and 1α,25(OH)_2_-3-epi-D_3_ in human keratinocytes. Keratinocytes in serum-free KGM (0.06 mM calcium) were seeded into 96-well plates and 24 h later the indicated metabolites (range 0.1–100 nM). After further 24 h, 1 µCi ^3^H-thymidine was applied to each well and its incorporation determined as described in Methods. Data are mean values (± SD) from a representative experiment out of two independent studies, each done in triplicates. For all experiments Student's unpaired t-test was performed and *p*-values were calculated between values obtained for 1α,25(OH)_2_D_3_ and 1α,25(OH)_2_-3-epi-D_3_ (* *p*<0.05, ** *p*<0.01, *** *p*<0.001).

The human transcriptional intermediary factor TIF2 coactivator (NCOA2) has been shown to interact with VDR [45]. The induced recruitment of TIF2 coactivator peptide bearing the 3rd LXXLL motif to the hVDR LBD was monitored in the presence of increasing concentrations of 1α,25(OH)_2_D_3_ or 1α,25(OH)_2_-3-epi-D_3_ using the luminescent oxygen channeling assays [46]. Our results show that EC_50_ value for both metabolites are in the lower nanomolar range, 1.2 and 2.5 nM for 1α,25(OH)_2_D_3_ and 1α,25(OH)_2_-3-epi-D_3_, respectively (Figure 2A).

### 1α,25(OH)_2_D_3_ and 1α,25(OH)_2_-3-epi-D_3_ induce expression of vitamin D target genes in human breast cancer (MCF-7) cells with similar potency

The transactivation potency of 1α,25(OH)_2_-3-epi-D_3_ has been reported for several VDR target genes in different model cell lines such as MG-63 or ROS17/2.8 osteosarcoma cells [24], [30]. While the transcriptional activity in MG-63 cells using a vitamin D-responsive element (VDRE) from human *osteocalcin* (−848/+10) and rat *CYP24* (−291/+9) gene promoters was lower upon stimulation with 1α,25(OH)_2_-3-epi-D_3_ compared to 1α,25(OH)_2_D_3_ [47], using 2xVDREs reporter from *CYP24* gene promoter in human melanoma G-361 cells comparable transcriptional activity was observed [48]. This response is mainly achieved in cells in which the 1α,25(OH)_2_-3-epi-D_3_ metabolite is produced [29]. We monitored the dose-dependent VDR induced transcriptional activity in human breast cancer cells (MCF-7) cells transfected with human *CYP24* promoter (−414 to −64) containing VDRE fused to reporter *luciferase* gene (Figure 2B). Here, we show that 1α,25(OH)_2_-3-epi-D_3_ is slightly less potent than 1α,25(OH)_2_D_3_ in directing transactivation assay as the EC_50_ induced by 1α,25(OH)_2_-3-epi-D_3_ is twice higher than that of 1α,25(OH)_2_D_3_ (5.9 nM vs 2.9 nM). This difference is in agreement with our results obtained from cell free coactivator peptide recruitment assays. Our transactivation assays show that the dose-dependent comparison between the 1α,25(OH)_2_-3-epi-D_3_ and 1α,25(OH)_2_D_3_ reveals that at 50% of the dose-response, the transcriptional activity of the 3-epimer is 65% of that obtained with 1α,25(OH)_2_D_3_. Statistical analysis revealed a significant correlation between both the induced-coactivator recruitment and transactivation assays (Pearson *r* = 0.961** and *r* = 0.986**, respectively), indicating the similarity in the course of the dose response curves for both 1α,25(OH)_2_D_3_ and 1α,25(OH)_2_-3-epi-D_3_. The reason for the discrepancy from the previously reported lower transactivation potential of 1α,25(OH)_2_-3-epi-D_3_ may have its origin in different *CYP24* promoter fragment used in our experiments. Although, the EMSA assays with nuclear extracts and *in vitro* translated full length VDR and RXR reported by Nakagawa *et al.* [47] showed decreased DNA complex formation of VDR-RXR heterodimer in the presence of 1α,25(OH)_2_-3-epi-D_3_ compared to 1α,25(OH)_2_D_3_, the same authors showed using two-hybrid system that the strength of VDR-RXR heterodimerization in presence of 10nM of the 3-epimer is 40% compared to that observed for 1α,25(OH)_2_D_3_.

### Cell specific effects of 1α,25(OH)_2_-3-epi-D_3_

The magnitude of 1α,25(OH)_2_-3-epi-D_3_-mediated specific biological outcomes versus that induced by 1α,25(OH)_2_D_3_ is cell line specific. As such, it is established based only on CD11b antigen positive cell numbers that 1α,25(OH)_2_-3-epi-D_3_ is biologically less potent than 1α,25(OH)_2_D_3_ in the human leukemia anti-proliferation and pro-differentiation cellular model (HL60), compared to 1α,25(OH)_2_D_3_ [47]. We monitor the precise dose-dependent study of 1α,25(OH)_2_-3-epi-D_3_ - directed HL60 cell anti-proliferation and differentiation by live cell enumeration and flow cytometry based on the expression of both CD11c and CD14 cell surface markers. In our experiments for both, 1α,25(OH)_2_D_3_ and 1α,25(OH)_2_-3-epi-D_3,_ only the saturating 100 nM concentration of ligand reduced the numbers of HL60 cells (Figure S1 and Methods S2). Although for 1α,25(OH)_2_-3-epi-D_3_ the related percentage of single positive or double CD11c/CD14 sub-populations was higher compared to that observed in control incubations, it was markedly reduced compared to that induced with 100 nM 1α,25(OH)_2_D_3_, consistent with the previous study [47].

Further, we hypothesized about the absence of the 1α,25(OH)_2_-3-epi-D_3_ signaling in HL60 cellular model and thus turned to characterize some of the biological properties of 1α,25(OH)_2_-3-epi-D_3_ in cells where it is produced [20], [21]. We first determined the kinetics of *CYP27B1-* and *CYP24A1*-catalyzed oxidation by monitoring the major lipophilic metabolites arising from a single pulse of ^3^H[26], [27]-25(OH)D_3_ at physiological concentration (20.6 nM). During the first two hours, we observed a rapid appearance of 1α,25(OH)_2_D_3_, from which at a slower rate the 3-epimer was irreversibly formed. In total, some 60 independent incubation experiments were performed on the kinetics of ^3^H[26], [27]-25(OH)D_3_ using primary keratinocytes from various donors and skin sites. In all experiments, highly comparable time course of 1α,25(OH)_2_D_3_ and 1α,25(OH)_2_-3-epi-D_3_ were recorded with 3-epimer exceeding 1α,25(OH)_2_D_3_ after longer incubation as shown in Figure 2C and in the detailed HPLC analysis in Figure S2 and Methods S2. Since the 1α,25(OH)_2_-3-epi-D_3_ is present steadily up to 5 h in rather high concentration in this tissue and the fact that the primary genomic effects of hVDR ligands are exerted in first hours suggested that primary keratinocytes may be a good cellular model to investigate the anti-proliferative actions of this metabolite. Therefore we determined the dose-dependent anti-proliferative effects of 1α,25(OH)_2_D_3_ and 1α,25(OH)_2_-3-epi-D_3_ using ^3^H-thymidine incorporation assay (Figure 2D), and found that the IC_50_ values for 1α,25(OH)_2_D_3_ and 1α,25(OH)_2_-3-epi-D_3_ were highly similar (41.4 and 66.1 nM, respectively) with no significant statistical difference (using unpaired *t*-test *p* = 0.074). In addition, we correlated the course of the anti-proliferation data between the two epimers and find a strong correlation (Pearson *r* = 0.940**) between them indicating the similar anti-proliferative activity for 1α,25(OH)_2_D_3_ and 1α,25(OH)_2_-3-epi-D_3_. The anti-proliferative effects of the two metabolites are comparable and they are in close agreement with our coactivator peptide recruitment (Figure 2A) and reporter gene assays (Figure 2B). Although in this assay we cannot totally exclude the possibility that the potential cell type specific difference in the function of the two natural ligands may be partly due to the accumulation of 1α,25(OH)_2_-3-epi-D_3_ in 1α,25(OH)_2_D_3_ treated cells with C3-epimerization ability leading to additive effect, we consider this accumulation process as a naturally occurring *in vivo* physiological event when 1α,25(OH)2D3 is present in these cells.

### Overall structure of the hVDR complexed to 1α,25(OH)_2_-3-epi-D_3_

**Figure 3:**
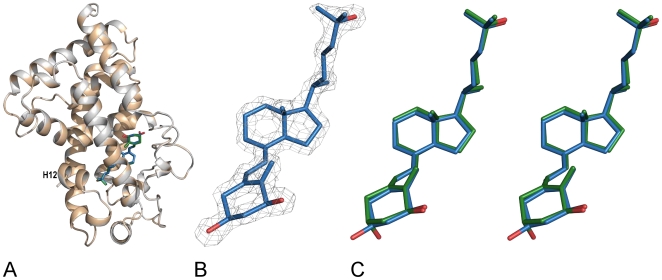
Overall structure of the VDR-1α,25(OH)_2_-3-epi-D_3_ and conformation of the bound ligand. (A) Superimposition of the hVDR LBD– 1α,25(OH)_2_-3-epi-D_3_ (blue) and the hVDR LBD–1α,25(OH)_2_D_3_ (white). The ligands are shown in stick representation in blue for the 1α,25(OH)_2_-3-epi-D_3_ and in green for the 1α,25(OH)_2_D_3_. (B) The 1α,25(OH)_2_-3-epi-D_3_ is shown in its Fo – Fc electron density omit map contoured at 3 σ. The ligand is shown in stick representation with carbon and oxygen atoms in blue and red, respectively. (C) Stereo view of the ligand 3D conformations of 1α,25(OH)_2_-3-epi-D_3_ (blue) and 1α,25(OH)_2_D_3_ (green) in their VDR ligand-binding pockets (LBP).

The mechanistic action of analogues of 1α,25(OH)_2_D_3_ is unveiled by the determination at high resolution of the crystal structure of their complexes with the VDR LBD [49]–[53]. We solved the crystal structure of the complex formed by 1α,25(OH)_2_-3-epi-D_3_ with the hVDR LBD mutant previously used to solve the structures of hVDR LBD in complexes with 1α,25(OH)_2_D_3_ or several synthetic agonists [49]–[54]. The crystal was isomorphous and the structure of hVDR LBD bound to 1α,25(OH)_2_-3-epi-D_3_ determined at a resolution of 1.9 Å (PDB ID: 3A78). The crystallographic data are summarized in Table S1. After refinement of the protein alone, the map showed an unambiguous electron density where to fit the ligand (Figure 3B). The complex formed by the hVDR LBD bound to 1α,25(OH)_2_-3-epi-D_3_ adopts the canonical active conformation as described in all previously reported agonist-bound nuclear receptor LBDs (Figure 3A). The conformation of the activation helix 12 is strictly maintained. When compared to the structure of hVDR LBD-1α,25(OH)_2_D_3_ complex, the atomic coordinates of hVDR LBD bound to 1α,25(OH)_2_-3-epi-D_3_ show very small root-mean-square deviation (RMSD) of 0.17 Å for all 255 Cα atoms, reflecting its high structural homology.

### Conformation of the 3α-epimer in the ligand-binding pocket of hVDR

The 1α,25(OH)_2_-3-epi-D_3_, is buried in the predominantly hydrophobic ligand-binding pocket (LBP) of the VDR. The conformation of the 3-epi-hydroxyl group does not modify the A-ring chair conformation of the ligand. Furthermore the seco B-, C-, D- rings, and the aliphatic side chain present conformations similar to those observed with 1α,25(OH)_2_D_3_ (Figure 3B and C).

**Figure 4:**
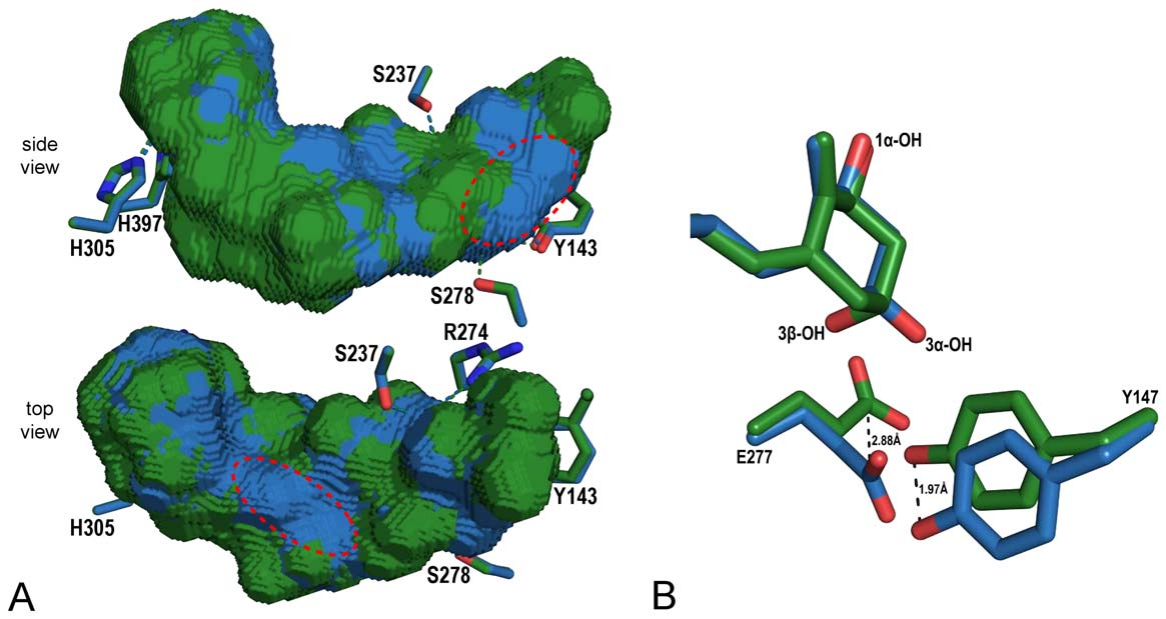
Adaptability of the hVDR LBP upon 1α,25(OH)_2_-3-epi-D_3_ binding. (A) The adaptation of the LBP is depicted by mesh representation of the superimposed LBP volumes calculated with Voidoo software. The green surface represent the LBP area where the 1α,25(OH)_2_D_3_ bound pocket is larger. The blue area represents similar increase but for 1α,25(OH)_2_-3-epi-D_3_ and the two main expanded regions are highlighted with red circles. (B) Adaptation of the residues Tyr147 and Glu277 in the LBP of the 1α,25(OH)_2_-3-epi-D_3_ hVDR complex. The distances between the ligand-specific positions of the residues are displayed in Å.

In the complexes of hVDR LBD bound to 1α,25(OH)_2_D_3_ versus 1α,25(OH)_2_-3-epi-D_3_, the distance between the C1-OH and the C25-OH groups varies from 13.1 Å to 12.7 Å and between the C3-OH and the C25-OH groups from 15.3 Å to 16.0 Å, respectively. The adaptation of the hVDR's LBP to different ligands can be described with the differential changes in the volumes of LBPs and bound ligands. In addition the parameter representing the % of LBP filling with ligand can provide useful information about the activity of ligand [55]. All these parameters are summarized in Table 1. Although the two diastereomer have the same molecular weight and differ only in the position of the C3-OH group, the 1α,25(OH)_2_-3-epi-D_3_ takes a slightly more compact conformation in the LBP. The graphical 0.2 Å mesh representation of the superimposed LPBs presented in Figure 4A and B show the surface area which is enlarged in case of 1α,25(OH)_2_D_3_ (in green) and the one enlarged in case of 1α,25(OH)_2_-3-epi-D_3_ bound hVDR LBP (in blue). This suggests that the hydrophobic residues lining the LBP are closer to the 3-epimer and may compensate for the canonical hydrogen bonds. We observed a notable adaptation with the displacement of the side chain of the residue Tyr147 by 2.0 Å compared to the 1α,25(OH)_2_D_3_ bound complex and the reorientation of the Glu277 side chain away from the 1α,25(OH)_2_-3-epi-D_3_ due to the α-position of the C3-OH group (Figure 4B). These specific rearrangements lead to a more compact conformation resulting in a 5% decrease in the volume of the LBP compared to 1α,25(OH)_2_D_3_.

**Table 1:** Volume of VDR ligands and their resulting LBPs.

Ligand	Ligand volume [Å^3^] *	Ligand volume [%]*	LBP volume [Å^3^]**	LBP volume [%]**	Filling of the LBP with ligand [%]
1α,25(OH)_2_D_3_	416.56	100.00	667.13	100.00	62.44
1α,25(OH)_2_-3-epi-D_3_	407.65	97.86	633.75	95.00	64.32

The absolute values in Å^3^ as well as relative values in reference to those of 1α,25(OH)_2_D_3_ (100%) are indicated. From these values the percent filling of the LBP with ligands was also calculated.

*and ** Connolly solvent accessible surfaces calculated by GRASP and Voidoo respectively The quality of the cubic grid spacing for the surface for both ligands and LBP = 0.5 Å.

### Specific interactions of the 1α,25(OH)_2_-3-epi-D_3_

**Figure 5:**
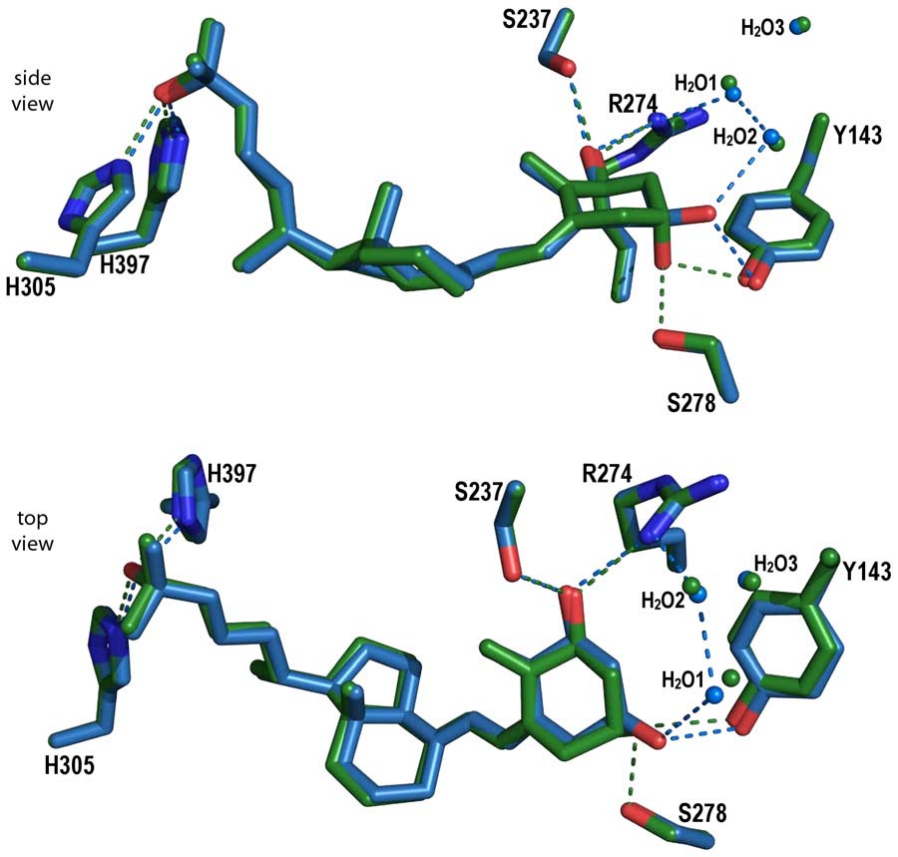
Specific interactions of 1α,25(OH)_2_-3-epi-D_3_ in the LBP of the hVDR. The ligands and residues in the superimposed structures are highlighted in color (1α,25(OH)_2_-3-epi-D_3_ in blue and in 1α,25(OH)_2_D_3_ green) and the important water molecules are represented with colored dots.

The hydrophobic and electrostatic interactions between the receptor and the ligand are similar between the two structures except around the C3-OH group. While the C1-OH and C25-OH display the canonical hydrogen bonds, the 3-epi-OH of 1α,25(OH)_2_-3-epi-D_3_ interacts through hydrogen bonding only with Tyr143 instead of interacting with both Tyr143 and Ser278 (Figure 5). A significant feature of the 1α,25(OH)_2_-3-epi-D_3_ is the compensation of the loss of interaction with Ser278 by a water-mediated hydrogen bond with the water molecule H_2_O1 (W1 in [50]). As such, the position of water H_2_O1 is moved 0.7 Å towards 1α,25(OH)_2_-3-epi-D_3_, thereby facilitating the specific water-mediated contacts. This water molecule is part of the network connecting another water molecule H_2_O2 to Arg274. All these water molecules are also present in the 1α,25(OH)_2_D_3_–hVDR complex [50]. The C3-OH hydrogen bonds have longer distances in the 3-epimer (3.0 Å instead of 2.8 Å with Tyr143 and 3.1 Å with the water molecule instead of 2.9 Å with Ser278). A study on the mutations of the residues forming the hydrogen bonds with the hydroxyl groups of 1α,25(OH)_2_-3-epi-D_3_ revealed that mutated residues contacting the 3-hydroxyl group are the less affected in term of activity. Mutation of Ser278 in Ala may result in a lower binding affinity for 1α,25(OH)_2_D_3_ [56] while showing a similar potency to activate the transcription [57], [56]. Due to the shift of the side chain of Tyr147, a hydrophobic interaction with this residue is lost in the 3-epimer structure. These structural data agree well with the lower binding affinity of this compound for VDR and to its induced biological activity.

In conclusion, we described a more effective synthesis of the highly stable 1α,25(OH)_2_-3-epi-D_3_, a natural metabolite. We have solved the crystal structure of hVDR LBD in complex with 1α,25(OH)_2_-3-epi-D_3_, which provides a mechanistic insight for the specific recognition of the two naturally occurring 3-epimers by hVDR. Indeed, the crystal structure reveals that the 3-epimer metabolite maintains the number of H-bonds by an alternative water-mediated interaction. In MCF-7 cells, the 1α,25(OH)_2_-3-epi-D_3_ on *CYP24* gene promoter retains significant transcriptional activity. In addition, the anti-proliferative action of 1α,25(OH)_2_-3-epi-D_3_ is cell specific and the IC_50_ values of 1α,25(OH)_2_D_3_ and 1α,25(OH)_2_-3-epi-D_3_ in primary keratinocytes are in the same nanomolar range. Therefore, we conclude that the C3-epimerization pathway produces an active metabolite with similar biochemical and biological properties to those of the 1α,25(OH)_2_D_3_. The physiological role of this pathway as the specific biological action of the 3-epimer remains unclear and needs further investigation. However, its high metabolic stability together with its significant biologic activity makes this natural metabolite an interesting ligand for clinical applications. Further study on its target specificity and selectivity is required to the design of selective analogues. Our new findings contribute to a better understanding at molecular level how natural metabolites of 1α,25(OH)_2_D_3_ lead to significant activity in biological systems.

## Materials and Methods

### Ligands

1α,25(OH)_2_D_3_ was purchased from Cayman Chemical (Tallinn, Estonia) and the synthesis of 1α,25(OH)_2_-3-epi-D_3_ is described in more details in the Methods S1. Additional ligands and reference compounds are described in Methods S2. IUPAC rules were used for the name of the compounds. In addition to NMR spectra (summarized in Methods S1), HPLC analysis was used to determine the purity (>95%) of the vitamin D analogues.

### Protein expression vectors for transactivation assays

Full-length cDNAs for human VDR [58] was subcloned into the T7/SV40 promoter-driven pSG5 expression vector (Stratagene, Heidelberg, Germany) and full-length cDNAs for green fluorescent protein (GFP) [59] was subcloned into parent vector resulting the pEGFP-C2 mammalian expression vector (Clontech Laboratories, Inc., USA).

### Luciferase reporter gene construct

The fragment of the proximal promoter region (−414 to −64) of the human *CYP24A1* gene was fused with the *thymidine kinase* promoter driving the firefly *luciferase* reporter gene [60].

### Coactivator peptide recruitment assays

Biochemical interaction between human VDR-LBD and the coactivator peptide in the presence of 1α,25(OH)_2_D_3_ or 1α,25(OH)_2_-3-epi-D_3_ was assayed using the AlphaScreen technology. The assay was performed in white opaque 384-well microplate (OptiPlate-384 Perkin Elmer) using a final volume of 15 µl containing final concentrations of 100 nM *E. coli*-expressed hexahistidine (6xHis)-tagged VDR-LBD protein, 20 nM of the human TIF2-3 biotinylated peptide (Btn-QEPVSPKKKENALLRYLLDKDDTKD), and 10 µg/ml of both AlphaLISA Ni^2+^-chelate acceptor beads and (AL108C) and AlphaScreen streptavidin coated donor beads (6760002S) in an assay buffer containing 50 mM MOPS pH = 7.4, 50 mM NaF, 50 mM CHAPS, and 100 µg/ml bovine serum albumin. Different concentrations of 1α,25(OH)_2_D_3_ or 1α,25(OH)_2_-3-epi-D_3_ dissolved in DMSO (maintained at a final concentration of 1%) were added as indicated. The experiment represents two independent measurements in triplicates for, which the mean and the S.D. of the mean was calculated.

### Transient transfections and luciferase reporter gene assays

MCF-7 cells were seeded into 24-well plates (100,000 cells/well) and grown overnight in phenol red-free Dulbecco's modified Eagle's medium (DMEM) supplemented with 10% charcoal-stripped fetal bovine serum (FCS) and 0.6 µg/ml insulin. Plasmid DNA containing liposomes were formed by incubating 40 ng of an expression vector for hVDR, 100 ng of reporter plasmid and 10 ng pEGF-C2 with Fugene 6 (Roche Diagnostics, Switzerland) transfection reagent according to the recommendation of the manufacturer for 15 min at room temperature. After dilution with 500 µl of phenol red-free DMEM, the liposomes were added to the cells. Phenol red-free DMEM supplemented with 500 µl of 20% charcoal-stripped FCS was added 4 h after transfection, in the presence of ligands or solvent. The cells were lysed 16 h after the onset of stimulation using reporter gene lysis buffer (Roche Diagnostics, Switzerland). The lysates were assayed for luciferase activity as recommended by the supplier (Perkin-Elmer, The Netherlands). The luciferase activities were normalized to GFP expression. Data represent one triplicate for which the mean and the S.D. of the mean was calculated.

### Data analysis for dose response curves

A non-linear curve fit was performed for the AlphaScreen and reporter gene assay experimental dose response data and from sigmoidal dose response curve then the EC_50_ values for the respective ligands were calculated using GraphPad Prism 4 (GraphPad Software Inc., San Diego, CA). The Student's unpaired *t*-test and Pearson correlation were performed with the SPSS software (SPSS Inc., version 14.0, Chicago, IL, USA).

### Keratinocyte cell cultures

Normal human keratinocytes were isolated from fresh adult skin obtained from surgery and immediately transported to the laboratory under sterile conditions. Isolation and culture under serum-free conditions and without a feeder layer followed a modified protocol as used by Bikle *et al* [11]. The isolated epidermis was incubated in a 0.25% trypsin solution for 45 min at 37°C. Thereafter, the cells were scraped off and put in 50 ml Hank's balanced salt solution (HBSS) containing 10% FCS to block further trypsin digestion and centrifuged at 2000 rpm/2 min. The resulting cell pellet was suspended in Keratinocyte Growth Medium (KGM, Clonetics Corp., San Diego), a defined serum-free medium at low (0.06 mM) calcium containing 0.1 ng/ml epidermal growth factor, 5 µg/ml insulin, 0.5 µg/ml hydrocortisone, bovine pituitary extract, antibiotics (gentamycin, amphothericin) gave the primary culture. After 24 h, the cells were incubated at 37°C in 95% air/5% CO_2_ and the attached cells were washed and provided with fresh KGM medium. The culture medium was changed every other day and the cells were passaged when they reached 80–90% confluency (usually 6–10 days after plating).

### Incubations of primary keratinocytes with ^3^H-25(OH)D_3_

Confluent human keratinocytes in 1 ml KGM and in 6-well plates were incubated in duplicates at 37°C with 20.6 nM ^3^H-25(OH)D_3_ (around 600 000 dpm/ml) for 1–23 h. Incubations were stopped with 1 ml methanol/well, the cells were scraped off, transferred to a test tube together with the supernatant and two washings (with 1 ml methanol and 0.8 ml water). Unmodified ^3^H-25(OH)D_3_ and most of the products were totally extracted from the combined solutions plus cell pellet according to the method of Bligh and Dyer [61] by three subsequent extractions with 2, 1 and 1 ml volumes of CHCl_3_ at room temperature. ^3^H-activity in the CHCl_3_-phase, in the water and total ^3^H-yield were determined. The combined CHCl_3_ extracts were then evaporated under argon at 35°C, the residues dissolved in 0.4 ml ethanol and an aliquot (containing around 250 000 dpm ^3^H-activity) subjected to HPLC-analysis (see Methods S2).

### ^3^H-Thymidine incorporation (anti-proliferation assay in primary keratinocytes)

Keratinocytes (second passage) in 200 µl KGM (low calcium) were plated in 96-well plates at an initial density of 10^4^ cells/well, kept 24 h at 37°C in an incubator with 95% air/5% CO_2_. Thereafter, the test compounds 1,25(OH)_2_D_3_ or its 3-epimer were added in 1 µl ethanol to give final concentrations ranging from 0 to 100 nM, each condition in triplicates. After further 24 h, 50 µl ^3^H-thymidine (1 µCi) were added and incubation continued for additional 7 h. Then, incubations were stopped by cell harvesting (Filtermate 196 Harvester, Packard-Canberra) and lysis: After removing the supernatant (see below), the adherent cells were released by 5 min treatment with 100 ml 0.125% trypsin in PBS at 37°C, harvested on a filterplate and washed 3 x with redistilled water. After drying the plates, their bottoms were sealed with a film and 50 µl scintillation cocktail (MicroScint O, Packard) were added. The whole plates were sealed with Packard Cover Film and ^3^H-activity counted on a Microplate Scintillation Counter (TopCount, Packard Canberra). To check whether proliferative (^3^H-thymidine incorporating) cells could have been shed off, the supernatants were soaked through a 96-well filterplate (Unifilter Plate GF/C) and 3 x washed with redistilled water: in all conditions, ^3^H-activity was undetectable on these filterplates (in order to roughly assess cell numbers and check for substance related morphological changes/toxic effects, photographs were taken prior to compound addition and immediately before harvesting.) Data - used as means ± SD – were normalized (incorporated ^3^H-activity sample vs. blank) and analyzed using the GRAFIT Erithacus 4.0.19 IC_50_ software.

### Protein purification and Crystallization

Purification and crystallization of the hVDR LBD complexed with 1α,25(OH)_2_-3-epi-D_3_ were performed as previously described [49]. The LBD of the hVDR (residues 118-427 Δ166-216) was cloned in pET-28b expression vector (Novagen) to obtain an N-terminal 6xHis fusion protein and was overproduced in E. coli BL21 (DE3) strain. Cells were grown in Luria Bertani medium and subsequently incubated for 6 h at 20°C with 1 mM isopropyl thio-β-D-galactoside. The protein purification included a metal affinity chromatography step on a Co^2+^-chelating resin (Clontech). The 6xHis tag was removed by thrombin digestion overnight at 4°C, and the protein was further purified by gel filtration on a Superdex S200 16/60. The sample buffer prior to protein concentration contained 10 mM Tris, pH = 7.5, 100 mM NaCl, and 10 mM dithiothreitol. The protein was concentrated to 3.5 mg/ml and incubated in the presence of a 1.5-fold molar excess of ligand. The purity and homogeneity of the protein were assessed by SDS-PAGE. The protein crystals were obtained at 4°C by vapor diffusion method using crystals of hVDR LBD-1α,25(OH)_2_D_3_ as microseeds. The reservoir solution contained 0.1 M MES and 1.4 M ammonium sulfate pH = 6.0.

### X-Ray data collection and structure determination

The crystal was mounted in fiber loop and flash cooled in liquid nitrogen after cryoprotection with a solution containing the reservoir plus 30% glycerol and 2% polyethylene glycol 400. Data collection from a single frozen crystal was performed at 100 K on the beamline ID29 of the European Synchrotron Radiation Facility (Grenoble, France). The crystal belongs to the orthorhombic space group P2_1_2_1_2_1_ with one monomer per asymmetric unit. Data were integrated and scaled using MOSFLM [62] (see statistics in Table S1). A rigid body refinement was used with the structure of the hVDR LBD complexed to 1α,25(OH)_2_D_3_ as a starting model. Refinement involved iterative cycles of manual building and refinement calculations. The programs Refmac [63] and COOT [64] were used throughout structure determination and refinement. The omit map from the refined atomic model of hVDR LBD was used to fit the ligand to its electron density, shown in Figure 2A. Individual B-atomic factors were refined isotropically. Solvent molecules were then placed according to unassigned peaks in the difference Fourier map. In the hVDR/1α,25(OH)_2_-3-epi-D_3_ complex, refined at 1.9 Å with no σ cutoff, the final model consists of residues 118-423 (Δ166–216), the ligand, two sulphate ions and 372 water molecules. According to PROCHECK [65] 92.6% of peptide lies in most favored regions and 7.4% in additional allowed regions. Data are summarized in Table S1. The volumes of the ligand-binding pockets and ligands were calculated as previously reported [49].

## Supporting Information

Figure S1 **Biological properties of 1α,25(OH)_2_D_3_ and 1α,25(OH)_2_-3-epi-D_3_ in HL60 cellular model.** (A) 1α,25(OH)2-3-epi-D3-mediated HL60 cell growth. 1α,25(OH)2D3 or 1α,25(OH)_2_-3-epi-D_3_-treated HL60 at 1 nM and 100 nM concentrations are counted. Data are presented as mean±S.D. of the mean (*, p<0.05; **, p<0.01; ***, p<0.001). (B) 1α,25(OH)_2_-3-epi-D_3_-mediated HL60 cell differentiation into monocyte-like cells. HL60 cells were treated with either ethanol or 1 nM and 100"nM concentration of 1α,25(OH)_2_D_3_ or 1α,25(OH)_2_-3-epi-D_3_. Cells were labeled with PElabeled anti-human CD11c and FITC-labeled anti-human CD14, and HL60 cell differentiation was estimated by the double-positive CD11c/CD14 population. Data are representative of three distinct experiments.
(PDF)

Figure S2 **Dominant production of the 1α,25(OH)_2_-3-epi-D_3_ in keratinocytes after 5 h.** HPLC profile of the CHCl3-extract from keratinocytes after 5 h incubation is shown. The amount of 1α,25(OH)_2_-3-epi-D_3_ (blue star) is the highest from all the metabolites detected with HPLC. The peak of 1α,25(OH)_2_D_3_ is highlighted with green star.
(PDF)

Table S1 **Data collection and refinement statistics.**
(PDF)

Methods S1 **Synthesis.**
(PDF)

Methods S2 (DOCX)

Scheme S1 **Retrosynthesis of 1b.**
(PDF)

Scheme S2 **Synthesis of enol triflate 3.** *Si* = TBS =  Si(*t*-Bu)(CH_3_)_2_.TBHP =  *t*-BuOOH (a) NaBH_4_, CeCl_3_·7H_2_O, MeOH, 0°C, 30 min. (b) TBHP, VO(acac)_2_, PhH, reflux, 30 min. (c) TBSCl, Im, DMF, rt, 12 h. (d) O_3_, MeOH-CH_2_Cl_2_, −78°C; Ac_2_O, Et_3_N, DMAP, −35°C to −8°C, 2 h; NaOAc, MeOH, 37°C, 12 h, (e) H_5_IO_6_, Et_2_O, rt, 2 h. (f) CBr_4_, Zn, Ph_3_P, CH_2_Cl_2_, rt, 40 min. (g) LDA, THF, −78°C, 1 h; *n*-BuLi, 15 min; 5-Cl-Py-_2_NTf_2_, −78°C to rt, 12 h.
(PDF)

Scheme S3 **Synthesis of metabolite 1.** TES  =  Si(CH_2_CH_3_)_3_. (a) (Ph_3_PCH_2_Br)Br, KO*t*-Bu, toluene, −5°C to rt, 1 h, 80%. (b) TESCl, Im, DMAP, DMF, rt, 3 h, 91%. (c) InCl_3_, *t*-BuLi, THF, −78°C to 0°C, 2 h. (d) 3, (Ph_3_P)_4_Pd, Et_3_N, THF, (dppf)PdCl_2_, 0°C to rt, 12 h. (e) HF·Py, Et_3_N, CH_2_Cl_2_, CH_3_CN, rt, 4 h, 58%.
(PDF)
