# Supplementary material for: 1α,25(OH)2-3-Epi-Vitamin D3, a Natural Physiological Metabolite of Vitamin D3: Its Synthesis, Biological Activity and Crystal Structure with Its Receptor
Source: PLoS One. 2011 Mar 31;6(3):e18124. doi: 10.1371/journal.pone.0018124 (PMC3069065; doi:10.1371/journal.pone.0018124)
Supplement: Figure S1 — Biological properties of 1α,25(OH)2D3 and 1α,25(OH)2-3-epi-D3 in HL60 cellular model. (A) 1α,25(OH)2-3-epi-D3-mediated HL60 cell growth. 1α,25(OH)2D3 or 1α,25(OH)2-3-epi-D3-treated HL60 at 1 nM and 100 nM concentrations are counted. Data are presented as mean±S.D. of the mean (*, p<0.05; **, p<0.01; ***, p<0.001). (B) 1α,25(OH)2-3-epi-D3-mediated HL60 cell differentiation into monocyte-like cells. HL60 cells were treated with either ethanol or 1 nM and 100"nM concentration of 1α,25(OH)2D3 or 1α,25(OH)2-3-epi-D3. Cells were labeled with PElabeled anti-human CD11c and FITC-labeled anti-human CD14, and HL60 cell differentiation was estimated by the double-positive CD11c/CD14 population. Data are representative of three distinct experiments. (PDF) [file pone.0018124.s001.pdf]

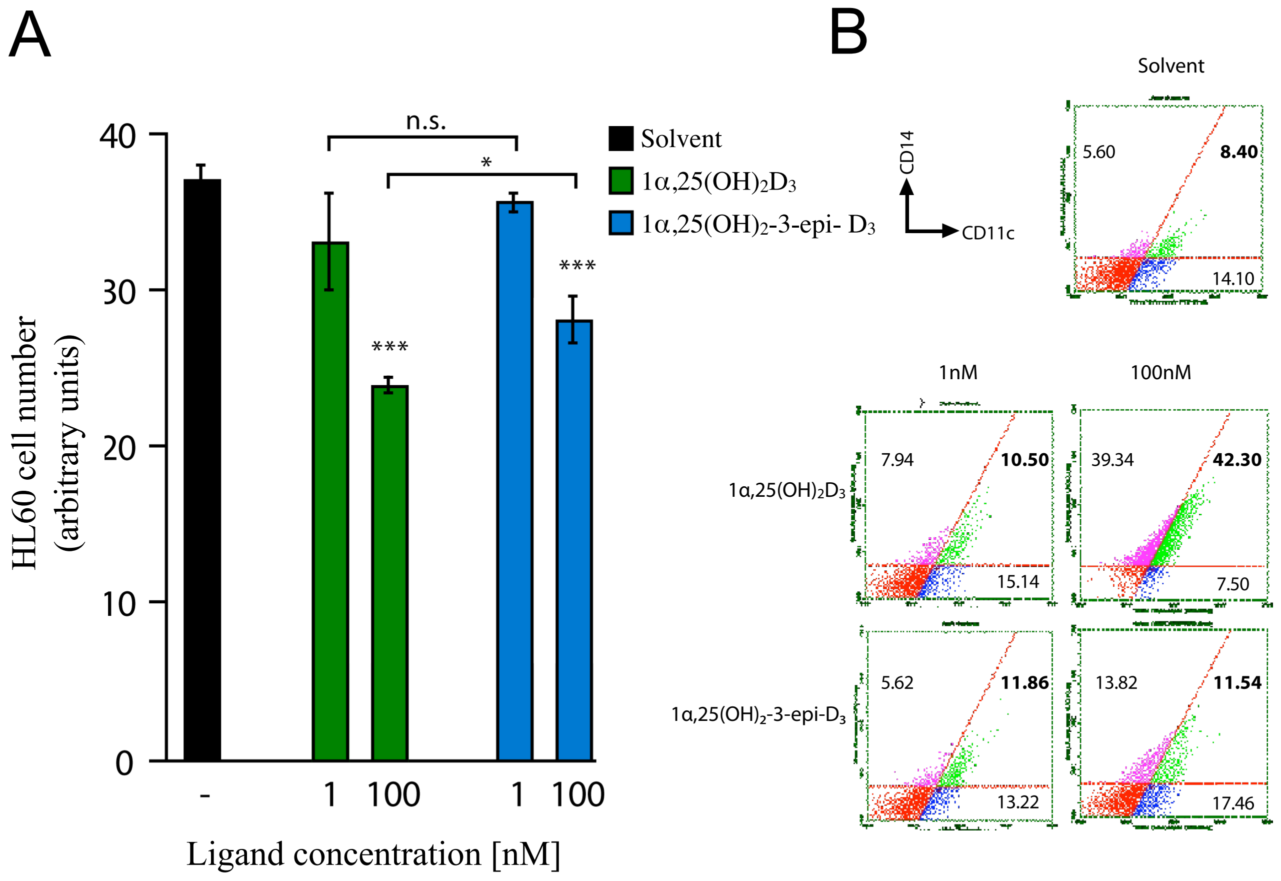

Figure S1. Biological properties of 1α,25$(OH)_2D_3$ and 1α,25$(OH)_2$-3-epi-D_3 in HL60 cellular model. (A) 1α,25$(OH)_2$-3-epi-D_3-mediated HL60 cell growth. 1α,25$(OH)_2D_3$ or 1α,25$(OH)_2$-3-epi-D_3-treated HL60 at 1 nM and 100 nM concentrations are counted. Data are presented as mean±S.D. of the mean (*, $p < 0.05$; **, $p < 0.01$; ***, $p < 0.001$). (B) 1α,25$(OH)_2$-3-epi-D_3-mediated HL60 cell differentiation into monocyte-like cells. HL60 cells were treated with either ethanol or 1nM and 100 nM concentration of 1α,25$(OH)_2D_3$ or 1α,25$(OH)_2$-3-epi-D_3. Cells were labeled with PE-labeled anti-human CD11c and FITC-labeled anti-human CD14, and HL60 cell differentiation was estimated by the double-positive CD11c/CD14 population. Data are representative of three distinct experiments.
