# Supplementary material for: 1α,25(OH)2-3-Epi-Vitamin D3, a Natural Physiological Metabolite of Vitamin D3: Its Synthesis, Biological Activity and Crystal Structure with Its Receptor
Source: PLoS One. 2011 Mar 31;6(3):e18124. doi: 10.1371/journal.pone.0018124 (PMC3069065; doi:10.1371/journal.pone.0018124)
Supplement: Figure S2 — Dominant production of the 1α,25(OH)2-3-epi-D3 in keratinocytes after 5 h. HPLC profile of the CHCl3-extract from keratinocytes after 5 h incubation is shown. The amount of 1α,25(OH)2-3-epi-D3 (blue star) is the highest from all the metabolites detected with HPLC. The peak of 1α,25(OH)2D3 is highlighted with green star. (PDF) [file pone.0018124.s002.pdf]

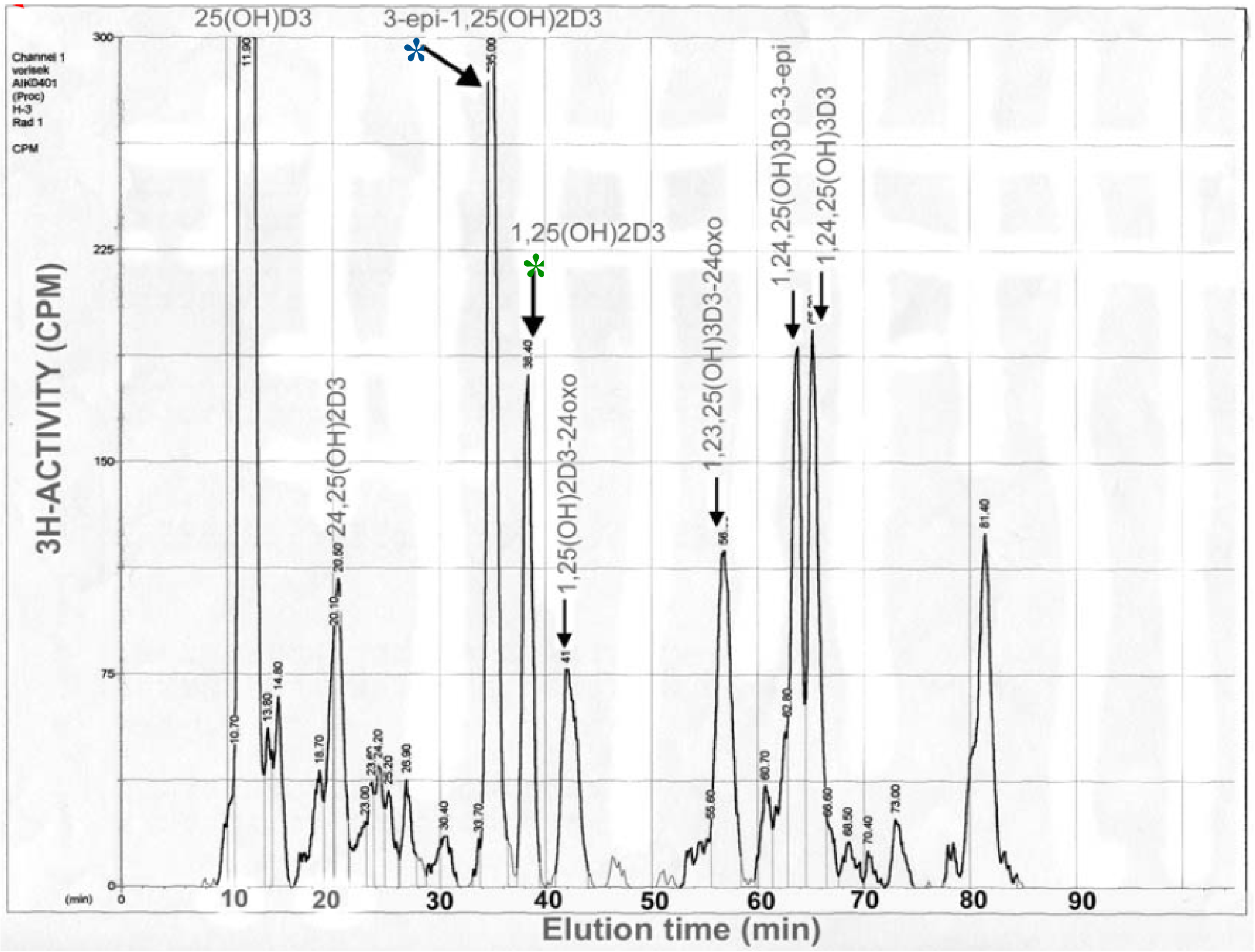

Figure S2. Dominant production of the 1α,25(OH)$_2$-3-epi-D$_3$ in keratinocytes after 5 h. HPLC profile of the $CHCl_3$-extract from keratinocytes after 5 h incubation is shown. The amount of 1α,25(OH)$_2$-3-epi-D$_3$ (blue star) is the highest from all the metabolites detected with HPLC. The peak of 1α,25(OH)$_2$D$_3$ is highlighted with green star.
