# Supplementary material for: 1α,25(OH)2-3-Epi-Vitamin D3, a Natural Physiological Metabolite of Vitamin D3: Its Synthesis, Biological Activity and Crystal Structure with Its Receptor
Source: PLoS One. 2011 Mar 31;6(3):e18124. doi: 10.1371/journal.pone.0018124 (PMC3069065; doi:10.1371/journal.pone.0018124)
Supplement: Table S1 — Data collection and refinement statistics. (PDF) [file pone.0018124.s003.pdf]

Supplementary Table 1. Data collection and refinement statistics.

	hVDR-LBD/1α,25$(OH)_2$-3epi-D_3
**Data processing**	
Resolution (Å)	48-1.9 (2.00-1.90)
Crystal space group	$P2_12_12_1$
Cell parameters (Å)	a = 44.9; b = 51.1; c = 132.2
Unique reflections	23145 (3268)
Mean redundancy	2.5 (2.4)
R_{sym} (%)[a]	7.8 (24.0)
Completeness (%)	94.3 (92.4)
Mean I/σ	11.3 (3.5)
Wilson B (Å^2)	15.1
**Refinement**	
Resolution (Å)	30-1.9
Number of protein atoms	2006
Number of ligand atoms	30
Number of water molecules	371
Number of sulfate ions	2
RMSD bond length (Å)	0.007
RMSD bond angles (˚)	1.130
R_{cryst} (%)[b]	17.2
R_{free} (%)[c]	20.3
Averaged B factor for nonhydrogen atoms (Å^2)	
Protein	12.9
Ligand	9.2
Sulfate ion	38.6
Water	30.2
Ramachandran plot (%)	
Core	92.6
Allowed	7.4

[a] R-sym: Σ|Ih-<Ih> | / ΣIh .

[b] Rcryst : Σ|Fo - Fc | / ΣFo, where Fo and Fc are the observed and calculated structure factor amplitudes respectively.

The [c] Rfreevalue was calculated from 5% of all the data that were not used in the refinement.
