# Supplementary material for: 1α,25(OH)2-3-Epi-Vitamin D3, a Natural Physiological Metabolite of Vitamin D3: Its Synthesis, Biological Activity and Crystal Structure with Its Receptor
Source: PLoS One. 2011 Mar 31;6(3):e18124. doi: 10.1371/journal.pone.0018124 (PMC3069065; doi:10.1371/journal.pone.0018124)

SUPPORTING INFORMATION - SYNTHESIS

CHEMISTRY

General. All reactions involving oxygen- or moisture-sensitive compounds were carried out under a dry Ar atmosphere using oven-dried or flame-dried glassware and standard syringe/septa techniques. Reaction temperatures refer to external bath temperatures. All dry solvents were distilled under Ar immediately prior to use. Tetrahydrofuran (THF), Et_2O and toluene were distilled from Na/benzophenone; CH_2Cl_2 was distilled from P_2O_5; *i*Pr_2NH, Et_3N, pyridine, and DMSO were distilled from CaH_2, DMF was dried over 4 Å molecular sieves. Solutions of *n*-BuLi in hexanes and *t*-BuLi in pentane were purchased from Aldrich. Liquid reagents or solutions of reagents were added by syringe or cannula. Organic extracts were dried over anhydrous Na_2SO_4, filtered and concentrated using a rotary evaporator at aspirator pressure (20-30 mm Hg). Reactions were monitored by thin-layer chromatography (TLC) using aluminium-backed MERCK 60 silica gel plates (0.2 mm thickness); the chromatograms were visualized first with ultraviolet light (254 nm) and then by immersion in solutions of ceric ammonium molybdate or *p*-anisaldehyde followed by heating with a hot gun. Flash column chromatography was performed with Merck silica gel 60 (230-400 mesh). All NMR spectra were measured with solutions in $CDCl_3$ unless otherwise stated. Chemical shifts are reported on the δ scale (ppm) downfield from tetramethylsilane (δ=0.0 ppm) using the residual solvent signal at δ=7.26 ppm (^{1}H) or δ=77 ppm (^{13}C) as internal standard. Coupling constants are reported in Hz. Data are reported as follows: chemical shift, multiplicity (s = singlet, d = doublet, t = triplet, q = quartet, m = multiplet, br = broad). Distortionless Enhancement by Polarization Transfer (DEPT) was used to assign carbon types. Melting points were measured in open capillarity tubes and are uncorrected. High resolution mass spectra (HRMS) were performed in a Micromas Instruments Autospec spectrometer. IR spectra were recorded as thin films on a silicon disc on a Bruker, model IFS-66V FT-IR spectrometer. Optical rotations were measured at 25ºC on a Jasco, model DIP-370 polarimeter in a 1 dm cell. [α] and *c* are given in $degcm^3g^{-1}dm^{-1}$ and gcm^{-3} respectively. HPLC purifications were performed on a Shimadzu preparative liquid chromatograph, Model LC-8A equipped with a UV-1 absorbance detector, using HPLC Phenomenex-Luna column (∅ 250 mm x 10 mm). Yields refer to chromatographically purified compounds, unless otherwise stated.

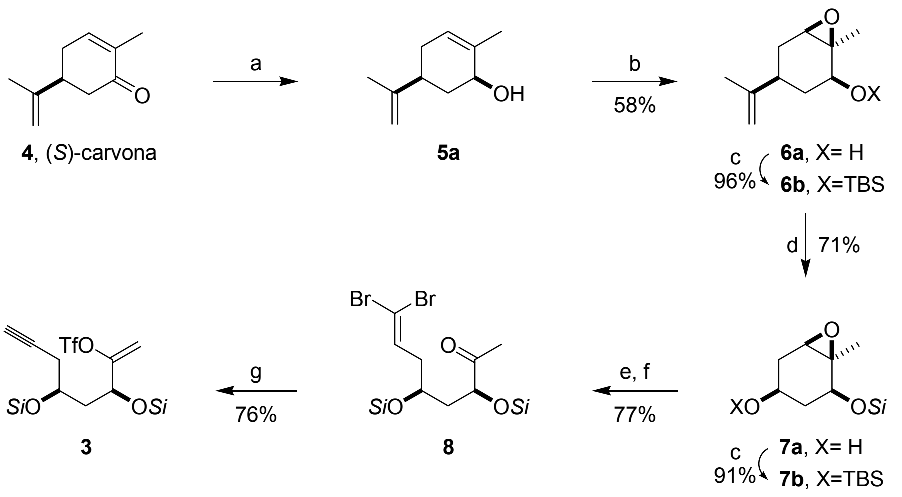

(a) $NaBH_4$, $CeCl_3 \cdot 7H_2O$, MeOH, 0 °C, 30 min. (b) TBHP, $VO(acac)_2$, PhH, reflux, 30 min. (c) TBSCl, Im, DMF, rt, 12 h. (d) O_3, $MeOH:CH_2Cl_2$, -78 °C; Ac_2O, Et_3N, DMAP, -35 °C → -8 °C, 2 h; NaOAc, MeOH, 37 °C, 12 h. (e) H_5IO_6, Et_2O, rt, 2 h. (f) CBr_4, Zn, Ph_3P CH_2Cl_2, rt, 40 min. (g) LDA, THF, -78 °C, 1 h; ^{n}BuLi, 15 min; 5-Cl-Py-2-NTf_2, -78 °C → rt, 12 h

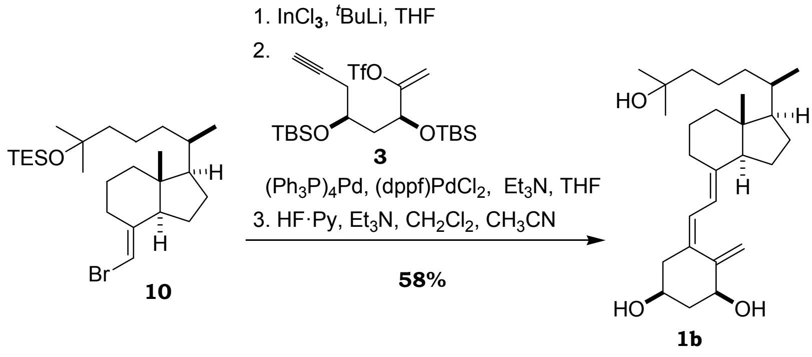

(1*S*/*R*,5*S*)-2-Methyl-5-(prop-1-en-2-yl)cyclohex-2-enol (5a)

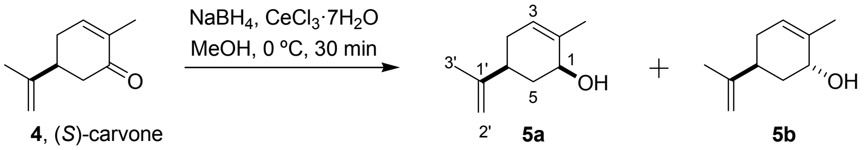

$NaBH_4$ (2.75 g, 72.6 mmol, 1.1 equiv) was added in small portions to a solution of (*S*)-carvone (10g, 66.6 mmol, 1 equiv) and $CeCl_3{\cdot}7H_2O$ (27 g, 72.6 mmol, 1.1 equiv) in dry MeOH (100 mL) at 0 ºC. After 30 min, the reaction was quenched by the dropwise addition of water. The mixture was concentrated to small volume, diluted with EtOAc (50 mL) and successively washed with aqueous HCl (30 mL, 5%), saturated NH_4Cl (20 mL) and H_2O (30 mL). The combined organic phase was dried, filtered and concentrated. The residue was used in the next reaction [10.3 g of **5**, mixture 9:1 according to ^{1}H-RMN, colorless oil, Rf= 0.25 (15% EtOAc-hexanes)].

^{1}H-RMN (250 MHz, $CDCl_3$): δ 5.48 (sa, 1H, H-3), 4.71 (s, 2H, CH_2-2'), 4.16 (s, 1H, OH), 2.30 -1.86 (m, 4H, CH_2-4, H-5 and H-6). 1.74 (s, 3H, CH_3-3'), 1.72 (s, 3H, CH_3C-2), 1.48 (c, 1H, J = 12.1 Hz, H-6). **^{13}C-RMN** (62.9 MHz, $CDCl_3$): δ 148.9 (=C, C-1'), 136.1 (=C, C-2), 123.8 (=CH, C-3), 109.1 (=CH_2, C-2'), 70.8 (CH, C-1), 40.4 (CH, C-5), 37.9 (CH_2, C-6), 30.9 (CH_2, C-4), 20.6 (CH_3, C-3'), 18.9 (CH_3, CH_3C-2).

(1*S*,2*S*,4*R*,6*R*)-1-methyl-4-(prop-1-en-2-yl)-7-oxa-bicyclo[4.1.0]heptan-2-ol (6a)

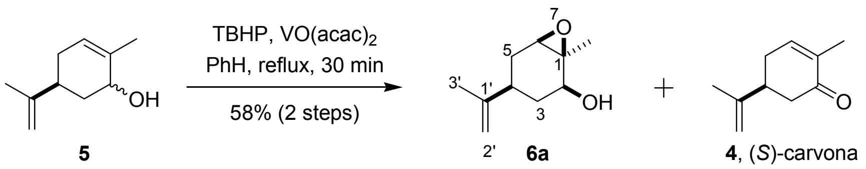

$VO(acac)_2$ (0.175 g, 0.66 mmol, 0.02 equiv) was added to a solution of the above alcohols **5** (5 g, 32.9 mmol, 1 equiv) in dry benzene (100 mL). The green solution refluxed. At the beginning of reflux, a solution of TBHP in decane (7.2 mL, 36.1 mmol, 5M, 1.1 equiv) was added dropwise. The initially green solution turned deep red and then yellow. After 30 min the heating bath was removed. Satured NH_4Cl (20 mL) was added at rt. The aqueous phase was extracted with Et_2O (2x50 mL). The combined organic phase was dried, filtered and concentrated. The residue was purified by flash chromatography (SiO_2, 4x10 cm, 10% EtOAc-hexanes) to give **6a** [3.2 g, 58%, colorless oil, Rf= 0.38 (20% EtOAc-hexanes)] and (*S*)-carvone [1.38 g, 28%, colorless oil, Rf= 0.625 (20% EtOAc-hexanes)].

^{1}H-RMN (400 MHz, CDCl$_3$): δ 4.64 (sa, 2H, CH$_2$-2'), 3.80 (dd, 1H, J_1 = 14.4 Hz, J_2 = 9.7 Hz, H-1), 3.11 (d, 1H, J = 4.9 Hz, H-5), 2.41 (sa, 1H, OH), 1.94 (m, 2H, H-4 and H-3), 1.71 (dm, 1H, J = 12.1 Hz, H-2), 1.63 (m, 1H, H-2), 1.62 (s, 3H, CH$_3$-3'), 1.40 (s, 3H, CH$_3$-19), 1.31 (c, 1H, J = 12.1 Hz, H-2). **^{13}C-RMN** (100.6 MHz, CDCl$_3$): δ 147.5 (C, C-1), 109.6 (CH$_2$, C-2'), 71.9 (CH, C-1), 62.1 (CH, C-5), 60.4 (C, C-10), 40.3 (CH, C-3), 33.7 (CH$_2$, C-2), 29.0 (CH$_2$, C-4), 20.0 (CH$_3$, C-3'), 19.1 (CH$_3$, C-19). **IR** (film, cm^{-1}): 3432 ($\nu_{O\text{-}H}$), 1645 ($\nu_{C=C}$). **EA**: calculado para: [$C_{10}H_{15}O_2$]: C(71.39), H(9.59); encontrado: C(71.76), H(9.94).

tert-butyldimethyl[(1*R*,2*S*,4*S*,6*R*)-1-methyl-4-(prop-1-en-2-yl)-7-oxa-bicyclo[4.1.0] heptan-2-yloxy]silane (6b)

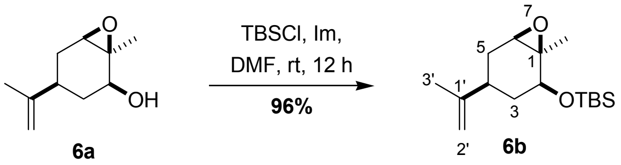

Imidazole (1.44 g, 21.2 mmol, 2 equiv) and TBSCl (2.4 g, 15.9 mmol, 1.5 equiv) were successively added to a solution of alcohol **6a** (3 g, 10.6 mmol, 1 equiv) in dry DMF (30 mL). After 12 h, the reaction was quenched by the addition of saturated NH_4Cl (50 mL). The aqueous phase was extracted with hexanes (3x40 mL). The combined organic phase was dried, filtered and concentrated. The residue was purified by flash chromatography (SiO_2, 4x14 cm, 2% EtOAc-hexanes) to give **6b** [4.8 g, 96%, solid, Rf= 0.87 (20% EtOAc-hexanes)].

^{1}H-RMN (250 MHz, CDCl$_3$): δ 4.68 (s, 2H, CH$_2$-2'), 3.91 (t, 1H, J = 7.4 Hz, H-2), 3.04 (d, 1H, J = 4.8 Hz, H-6), 1.67 (s, 3H, CH$_3$-3'), 1.36 (s, 3H, CH$_3$C-1), 0.91 (s, 9H, Me$_3$C-Si), 0.10 (s, 3H, Me-Si), 0.08 (s, 3H, Me-Si). **^{13}C-RMN** (62.9 MHz, CDCl$_3$): δ 148.1 (=C, C-1'), 109.6 (CH, C-2'), 73.2 (CH, C-2), 60.9 (CH, C-6), 60.2 (C, C-1), 40.6 (CH, C-4), 33.7 (CH$_2$, C-3), 29.1 (CH$_2$, C-5), 25.8 (CH$_3$, Me$_3$C-Si), 20.0 (CH$_3$, C-3'), 19.7 (CH$_3$, CH$_3$C-1), 18.1 (C, C-Si), -4.0 (CH$_3$, Me-Si), -4.7 (CH$_3$, Me-Si). **IR** (film, cm^{-1}): 1647 ($\nu_{C=C}$). **HMRS** ([CI]$^+$): calculated for: [$C_{16}H_{31}O_2Si$]$^+$ ([M+H]$^+$): 283.2093; found: 283.2093.

(1*R*,3*S*,5*S*,6*R*)-5-(*tert*-butyldimethylsilyloxy)-6-methyl-7-oxa-bicyclo[4.1.0]heptan-3-ol (7a)

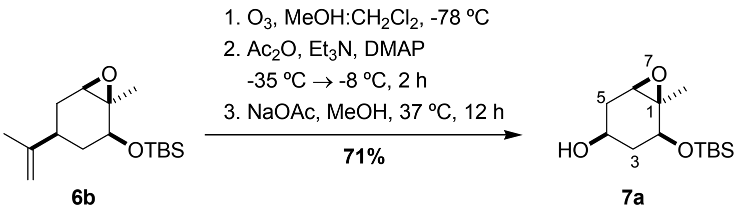

A stream of O_3/O_2 (0.7 bar, 0.1 NL/h, 50 w) was bubbled through a -78 ºC cooled solution of compound **6b** (2 g, 7.08 mmol, 1 equiv) in dry MeOH (5 mL) and dry CH_2Cl_2 (20 mL) until the solution turned blue (25 min). The excess of O_3 was removed with a flow of argon for 30 min. The reaction mixture was allowed to reach rt (40 min) under a slow flow of argon and then cooled to -35 ºC. After 10 min, Et_3N (9.7 mL, 70.8 mmol, 10 equiv) and DMAP (0.17 g, 1.41 mmol, 0.2 equiv) were successively and slowly added. Once DMAP has dissolved, Ac_2O (6.7 mL, 70.8 mmol, 10 equiv, freshly distilled from P_2O_5 under argon) was added. The reaction mixture was allowed to reach -8 ºC and stirred for 2 h. The reaction was quenched by the slow addition of MeOH (5 mL). The mixture was stirred at rt for 5 min, diluted with EtOAc (20 mL) and successively washed with an aqueous citric acid (2x20 mL, 10%) and saturated $NaHCO_3$ (2x15 mL). The combined organic phase was dried, filtered and concentrated. The residue was dissolved in MeOH (40 mL). Then, NaOAc (0.120 g, 1.41 mmol, 0.2 equiv) was added. The mixture was heated at 37 ºC for 12 h and then concentrated to half volume. The residue was dissolved in EtOAc (20 mL) and washed with saturated NH_4Cl (20 mL). The aqueous phase was extracted with EtOAc (2x20 mL). The combined organic phase was dried, filtered and concentrated. The residue was purified by flash chromatography (SiO_2, 3x12 cm, 20%EtOAc-hexanes) to give alcohol **7a** [1.30 g, 71%, colorless oil, Rf= 0.50 (50%EtOAc-hexanes)].

^{1}H-RMN (250 MHz, $CDCl_3$): δ 3.98 (dd, 1H, J_1 = 9.1 Hz, J_2 = 5.5 Hz, H-5), 3.65 (m, 1H, H-3), 2.97 (d, 1H, J = 4.4 Hz, H-1), 2.18 (ddd, 1H, J_1 = 14.8 Hz, J_2 = 6.2 Hz, J_3 = 4.6 Hz, H-2), 2.00 (d, 1H, J =5.5 Hz, OH), 1.80 (dd, 1H, J_1 = 14.8 Hz, J_2 = 8.9 Hz, H-4), 1.74 (1H, m, H-2), 1.63 (dd, 1H, J_1 = 10.6 Hz, J_2 = 9.3 Hz, H-4), 1.34 (s, 3H, CH_3C-6), 0.90 (s, 9H, Me_3C-Si), 0.10 (s, 3H, Me-Si), 0.09 (s, 3H, Me-Si). **^{13}C-RMN** (62.9 MHz, $CDCl_3$): δ 70.6 (CH, C-1), 65.5 (CH, C-3), 60.1 (CH, C-1), 58.6 (C, C-6), 38.0 (CH_2, C-4), 33.2 (CH_2, C-2), 25.8 (3xCH_3, Me_3C-Si), 19.7 (CH_3, CH_3C-6), 18.0 (C, C-Si), -4.2 (CH_3, Me-Si), -4.8 (CH_3, Me-Si). **IR** (film, cm^{-1}): 3422 ($\nu_{O\text{-}H}$). **HMRS** ($[CI]^+$): calculated for: $[C_{13}H_{27}O_3Si]^+$ ($[M+H]^+$): 259.1729; found: 259.1731.

(1*R*,2*S*,4*S*,6*R*)-2,4-bis(*tert*-butyldimethylsilyloxy)-1-methyl-7-oxa-bicyclo[4.1.0] heptanes (7b)

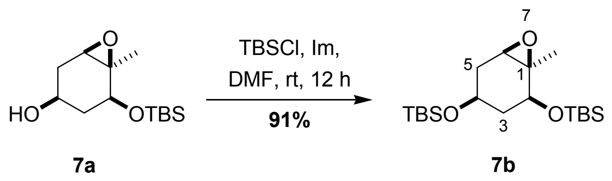

Imidazole (0.9 g, 13.2 mmol, 3 equiv) and TBSCl (1 g, 6.6 mmol, 1.5 equiv) were successively added to a solution of alcohol **7a** (1.14 g, 4.4 mmol, 1 equiv) in dry DMF (10 mL). After 12 h, the reaction was quenched by the addition of ice pieces and saturated NH_4Cl (50 mL). The aqueous phase was extracted with hexanes (3x20 mL). The combined organic phase was dried, filtered and concentrated in vacuo. The residue was purified by flash chromatography (SiO_2, 2x14 cm, hexanes) to give **7b** [1.49 g, 91%, colorless oil, Rf= 0.62 (10% EtOAc-hexanes)].

^{1}H-RMN (300 MHz, $CDCl_3$): δ 3.91 (dd, 1H, J_1 = 10.3 Hz, J_2 = 5.6 Hz, H-2), 3.56 (tdd, 1H, J_1 = 11.0 Hz, J_2 = 6.9 Hz, J_3 = 4.4 Hz, H-4), 2.87 (d, 1H, J = 5.1 Hz, H-6), 2.09 (ddd, 1H, J_1 = 14.7 Hz, J_2 = 6.9 Hz, J_3 = 5.2 Hz, H-5), 1.74 (dd, 1H, J_1 = 14.9 Hz, J_2 = 10.0 Hz, H-5), 1.66 (m, 1H, H-3), 1.64 (m, 1H, H-3), 1.32 (s, 3H, CH_3C-1), 0.91 (s, 9H, Me_3C-Si), 0.86 (s, 9H, Me_3C-Si), 0.10 (s, 3H, Me-Si), 0.08 (s, 3H, Me-Si), 0.033 (s, 3H, Me-Si), 0.030 (s, 3H, Me-Si). **^{13}C-RMN** (75 MHz, $CDCl_3$): δ 71.2 (CH, C-2), 66.4 (CH, C-4), 60.1 (C, C-1), 58.2 (CH, C-6), 38.5 (CH_2, C-3), 34.0 (CH_2, C-5), 25.8 (6xCH_3, 2xMe_3C-Si), 19.2 (CH_3, CH_3C-1), 18.1 (2xC, 2xC-Si), -4.2 (CH_3, Me-Si), -4.7 (3xCH_3, Me-Si). **EA:** calculated for: [$C_{19}H_{41}O_3Si_2$]: C(61.23), H(10.82); found: C(61.56), H(11.19).

(3*R*,5*S*)-3,5-bis(*tert*-butyldimethylsilyloxy)-6-oxoheptanal (8a)

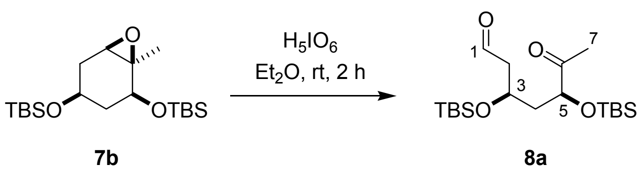

H_5IO_6 (2.77 g, 12.1 mmol, 3 equiv) was added to a solution of epoxide **7b** (1.51 g, 4.05 mmol, 1 equiv) in dry Et_2O (20 mL). After 2 h, the reaction mixture was poured onto an aqueous saturated solution of $Na_2S_2O_3$ (50 mL). The aqueous phase was extracted with Et_2O (3x20 mL). The combined organic phase was dried, filtered and concentrated to give aldehyde **8a** [1.5 g, Rf= 0.37 (10% EtOAc-hexanes)], which was immediately used in the next experiment.

^{1}H-RMN (250 MHz, $CDCl_3$): δ 9.75 (t, 1H, J = 2.2 Hz, H-1), 4.28 (q, 1H, J = 5.9 Hz, H-3), 4.06 (t, 1H, J = 6.4 Hz, H-5), 2.61 (ddd, 1H, J_1 = 16.1 Hz, J_2 = 5.2 Hz, J_3 =1.9 Hz, H-2), 2.51 (ddd, 1H, J_1 = 16.1 Hz, J_2 = 6.6 Hz, J_3 = 2.6 Hz, H-2), 2.13 (s, 3H, C-7), 1.86 (dd, 1H, J_1 = 14.1 Hz, J_2 = 6.6 Hz, H-4), 1.75 (dd, 1H, J_1 = 14.1 Hz, J_2 = 6.4 Hz, H-4), 0.88 (s, 9H, Me_3C-Si), 0.83 (s, 9H, Me_3C-Si), 0.06 (s, 3H, Me-Si), 0.03 (s, 9H, 3xMe-Si). **^{13}C-RMN** (62.9 MHz, $CDCl_3$): δ 210.7 (C=O, C-6), 201.3 (HC=O, C-1), 75.6 (CH, C-5), 64.7 (CH, C-3), 50.6 (CH_2, C-2), 42.1 (CH_2, C-4), 25.6 (6xCH_3, 2xMe_3C-Si), 25.0 (CH_3, C-7), 17.9 (2xC, 2xC-Si), -4.7 (CH_3, Me-Si), -5.0 (CH_3, Me-Si), -5.2 (2xCH_3, Me_2-Si).

(3*S*,5*S*)-8,8-dibromo-3,5-bis(*tert*-butyldimethylsilyloxy)oct-7-en-2-one (8b)

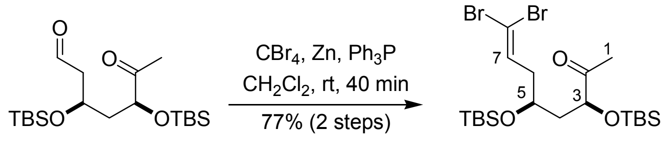

CBr_4 (4.02 g, 12.15 mmol, 3 equiv) was added to a suspension of Ph_3P (3.18 g, 12.15 mmol, 3 equiv) and Zn (0.8 g, 12.15 mmol, 3 equiv) in CH_2Cl_2 (40 mL) at 0 ºC. After 5 min, the cooling bath was removed and the reaction mixture was stirred at rt for 2 h. The color changed from green to deep red. A solution of aldehyde **8a** (1.52 g, 4.05 mmol, 1 equiv) in CH_2Cl_2 (10 mL) was added via cannula. After 45 min, the reaction mixture was filtered through a path of silicagel (elution with hexanes and 20% EtOAc-hexanes). After concentration, the residue was purified by flash chromatography (SiO_2, 3.5x15 cm, 2% EtOAc-hexanes) to give dibromide **8b** [1.69 g, **77%** 2 steps, colorless oil, Rf= 0.75 (4% EtOAc-hexanes)].

^{1}H-RMN (250 MHz, $CDCl_3$): δ 6.45 (t, 1H, J = 7.2 Hz, H-7), 4.09 (t, 1H, J = 6.5 Hz, H-3), 3.95 (q, 1H, J = 5.7 Hz, H-5), 2.30 (m, 2H, CH_2-6), 2.15 (s, 3H, CH_3-1), 1.79 (dd, 1H, J_1 =13.9 Hz, J_2 = 6.3 Hz, H-4), 1.69 (dd, 1H, J_1 = 13.9 Hz, J_2 = 6.3 Hz, H-4), 0.92 (s, 9H, Me_3C-Si), 0.88 (s, 9H, Me_3C-Si), 0.07 (s, 9H, 3xMe-Si), 0.06 (s, 3H, Me-Si). **^{13}C-RMN** (62.9 MHz, $CDCl_3$): 210.8 (C=O, C-2), 135.0 (=CH, C-7), 90.1 (=C, C-8), 75.7 (CH, C-3), 67.0 (CH, C-5), 41.7 (CH_2, C-4), 40.4 (CH_2, C-6), 25.8 (3xCH_3, Me_3C-Si), 25.7 (3xCH_3, Me_3C-Si), 25.2 (CH_3, C-1), 18.0 (C, C-Si), -4.60 (CH_3, Me-Si), -4.7 (CH_3, Me-Si), -4.86 (CH_3, Me-Si), -4.92 (CH_3, Me-Si). **IR** (film, cm^{-1}): 1716 ($\nu_{C=O}$). **HMRS** ([CI]$^+$): calculated for: $[^{12}C_{20}H_{41}O_3Si^{79}Br^{81}Br]^+$ ($[M+H])^+$: 545.0974; found: 545.0969.

(3S,5S)-3,5-bis(*tert*-butyldimethylsilyloxy)oct-1-en-7-yn-2-yl trifluoromethanesulfonate (3)

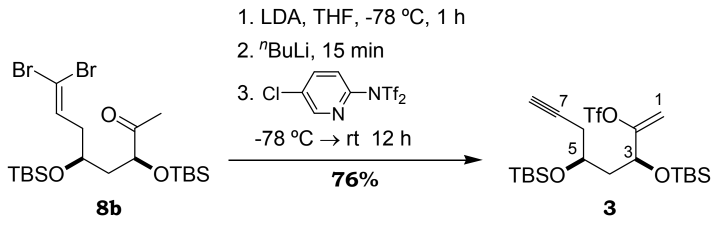

A freshly prepared solution of LiN(*i*-Pr)$_2$ in THF (1.1 mL, 9.4 mmol, 0.9M, 3 equiv) was added dropwise to a solution of the dibromide **8b** (1.7 g, 3.12 mmol, 1 equiv) in THF (20 mL) at -78 ºC. After stirring for 1 h, a solution of *n*-BuLi in hexanes (0.3 mL, 0.75 mmol, 2.5M, 0.3 equiv) was added. After 15 min, *N*-(5-chloro-2-pyridyl)bis(trifluoromethanesulfonimide) (3.7 g, 9.4 mmol, 3 equiv) was added at once. The reaction mixture was allowed to reach rt over 12 h. The reaction was quenched by the addition of saturated NaCl (10 mL). The aqueous phase was extracted with Et_2O (2x20 mL). The combined organic phase was dried, filtered and concentrated. The residue was purified by flash chromatography (SiO_2, 3x12 cm, hexanes) to give enol triflate **3** [1.2 g, 76%, colorless oil, Rf= 0.70 (2% Et_2O-hexanes)].

^{1}H-RMN (250 MHz, $CDCl_3$): δ 5.21 (d, 1H, J = 3.5 Hz, H-1), 5.19 (d, 1H, J = 3.5 Hz, H-1), 4.39 (t, 1H, J = 6.3 Hz, H-3), 3.86 (q, 1H, J = 5.8 Hz, H-5), 2.38 (m, 2H, CH_2-6), 2.07 (dt, 1H, J_1 = 14.1 Hz, J_2 = 6.0 Hz, H-4), 1.99 (t, 1H, J = 2.6 Hz, H-8), 1.85 (dt, 1H, J_1 = 14.1 Hz, J_2 = 6.3 Hz, H-4), 0.92 (s, 9H, Me_3C-Si), 0.89 (s, 9H, Me_3C-Si), 0.11 (CH_3, Me-Si), 0.09 (CH_3, Me-Si), 0.08 (CH_3, Me-Si), 0.06 (CH_3, Me-Si). **^{13}C-RMN** (62.9 MHz, $CDCl_3$): 156.4 (=C, C-2), 104.4 (=CH_2, C-1), 80.7 (≡C, C-7) 70.6 (≡CH, C-8), 69.4 (CH, C-3), 67.4 (CH, C-1), 41.6 (CH_2, C-4), 27.4 (CH_2, C-6), 25.74 (CH_3, Me_3C-Si), 25.70 (CH_3, Me_3C-Si), 18.05 (C, C-Si), 17.99 (C, C-Si), -4.3 (CH_3, Me-Si), -4.8 (CH_3, Me-Si), -4.9 (CH_3, Me-Si), -5.1 (CH_3, Me-Si). **IR** (film, cm^{-1}): 3315 ($\nu_{C\equiv C}$), 1668 ($\nu_{C=C}$). **HMRS** ([CI]$^+$): calculated for: $[C_{21}H_{40}O_2Si_2S]^+$ ($[M+H]^+$): 517.2087; found: 517.2093.

1α,25-dihidroxi-3-*epi*-vitamina D$_3$ (1b)

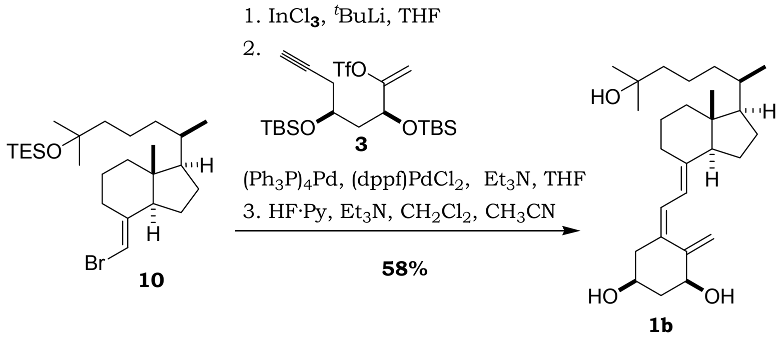

A solution of $InCl_3$ (0.230 mL, 0.106 mmol, 0.47 M, 0.5 equiv) in dry THF was added via syringe to a solution of bromide **10** (100 mg, 0.212 mmol, 1 equiv) in dry THF (2 mL). The colorless mixture was cooled at -78 ºC and then a solution of *t*-BuLi in pentane was added dropwise (0.450 mL, 0.765 mmol, 1.7M, 3.6 equiv). After 1 h, the -78 ºC cooling bath was replaced by and ice-H_2O bath. The slightly yellow solution was stirred for 1 h. A solution of enol triflate **10** (0.068 g, 0.130 mmol, 0.5 equiv), $(Ph_3P)_4Pd$ (15 mg, 0.013 mmol, 6 mol%) and Et_3N (0.1 mL, 0.721 mmol, 3.4 equiv) in dry THF (1 mL), and (dppf)$PdCl_2$ (10 mg, 0.013 mmol, 6 mol%) were successively added. The reaction mixture was stirred in the dark for 5 min at 0 ºC and then at rt for 12 h. The reaction was quenched by the addition of satured NH_4Cl (3 mL). The aqueous phase was extracted with Et_2O (3x3 mL). The combined organic phase was dried, filtered and concentrated in vacuo. The residue was dissolved in a mixture of dry CH_2Cl_2 (1 mL), CH_3CN (2 mL) and Et_3N (1 mL). HF·Py complex (0.2 mL, 70:30 HF·Py) was added. The mixture was stirred at rt for 1 h. The reaction was quenched by the addition of saturated $NaHCO_3$ (5 mL). The aqueous phase was extracted with EtOAc (3x10 mL). The combined organic phase was dried, filtered and concentrated in vacuo. The residue was purified by flash chromatography (SiO_2, 1.5x10 cm, 50% EtOAc-hexanes) and then by HPLC (Phenomenex, Silica(2), 250x1.2 mm, 20% *i*-PrOH-hexanes) to give **1b** [0.032 g, 58%, white solid, Rf= 0.45 (80% EtOAc-hexanes)].

^{1}H-RMN (500 MHz, $CDCl_3$): δ 6.43 (dd, 1H, J = 11.2 Hz, H-6), 6.02 (dd, 1H, J = 11.3 Hz, H-7), 5.29 (s, 1H, H-19), 5.00 (d, 1H, J = 1.7 Hz, H-19), 4.31 (d, 1H, J = 4.5 Hz, H-1), 4.05 (m, 1H, H-3), 2.84 (dm, 1H, J = 12.5 Hz, H-9), 2.56 (dd, J_1 = 13.5 Hz, J_2 = 2.3 Hz, H-4α), 2.43 (dd, 1H, J_1 = 13.4 Hz, J_2 = 5.7 Hz, H-4β), 2.07 (dt, 1H, J_1 = 13.5 Hz, J_2 = 3.63 Hz, H-12), 1.21 (s, 6H, CH_3-26 and CH_3-27), 0.94 (d, 3H, J = 6.5 Hz, CH_3-21), 0.54 (s, 3H, CH_3-18). **^{13}C-RMN** (125.7 MHz, $CDCl_3$): δ 147.2 (=C, C-10), 143.2 (=C, C-8), 131.6 (=C, C-25), 125.6 (=CH, C-6), 117.0 (=CH, C-7), 112.9 (=CH_2, C-19), 73.1 (CH, C-1), 71.1 (C, C-25), 68.2 (CH, C-3), 56.5 (CH, C-17), 56.3 (CH, C-14), 45.9 (C, C-13), 45.5 (CH, C-2), 44.4 (CH_2, C-4), 40.7 (CH_2), 40.5 (CH_2), 36.4 (CH_2), 36.1 (CH, C-20), 29.4 (CH_3, C-27), 29.2 (CH_3, C-26), 29.1 (CH_2, C-9), 27.7 (CH_2), 23.5 (CH_2), 22.2 (CH_2), 20.8 (CH_2), 18.8 (CH_3, C-21), 12.0 (CH_3, C-18). **IR** (film, cm^{-1}): 3365 ($\nu_{O\text{-}H}$), 1713, 1631 ($\nu_{C=C}$). **HMRS** ($[CI]^+$): calculated for: $[C_{27}H_{45}O_3]^+$ ($[M+H]^+$): 417.3369; found: 417.3375.

NMR Spectra of compounds

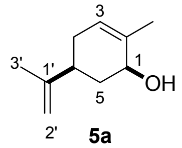

5a

[1]**H-RMN** (250 MHz, $CDCl_3$): δ 5.48 (sa, 1H, H-3), 4.71 (s, 2H, CH_2-2’), 4.16 (s, 1H, OH), 2.30 -1.86 (m, 4H, CH_2-4, H-5 and H-6). 1.74 (s, 3H, CH_3-3’), 1.72 (s, 3H, CH_3C-2), 1.48 (c, 1H, *J* = 12.1 Hz, H-6).

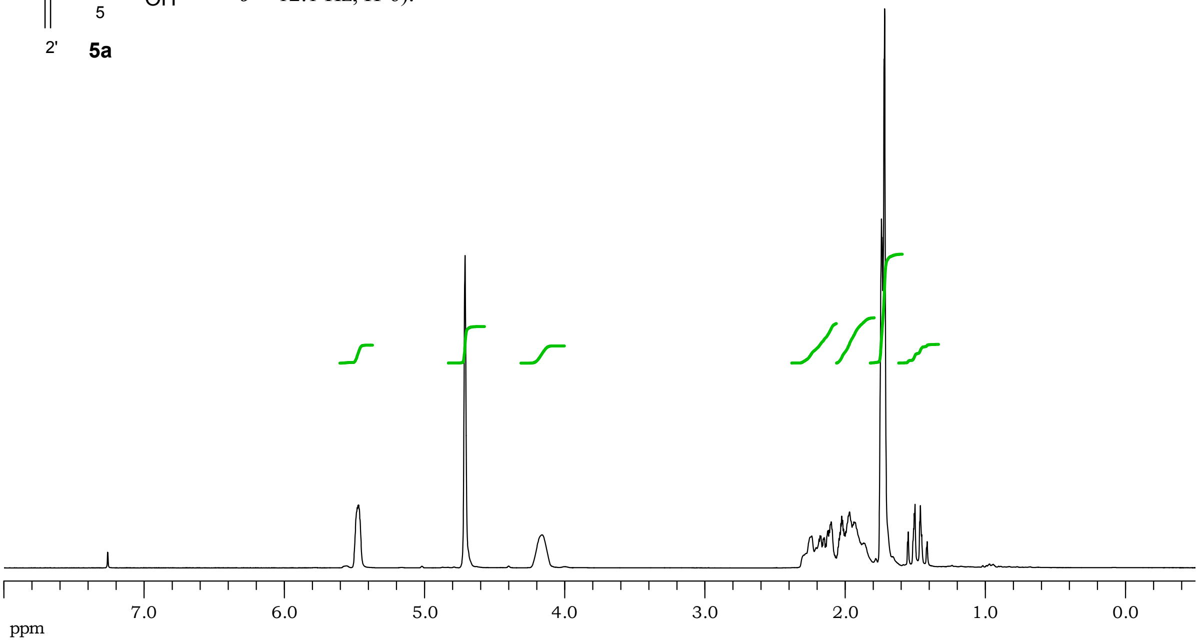

[13]**C-RMN** (62.9 MHz, $CDCl_3$): δ 148.9 (=C, C-1’), 136.1 (=C, C-2), 123.8 (=CH, C-3), 109.1 (=CH_2, C-2’), 70.8 (CH, C-1), 40.4 (CH, C-5), 37.9 (CH_2, C-6), 30.9 (CH_2, C-4), 20.6 (CH_3, C-3’), 18.9 (CH_3, CH_3C-2).

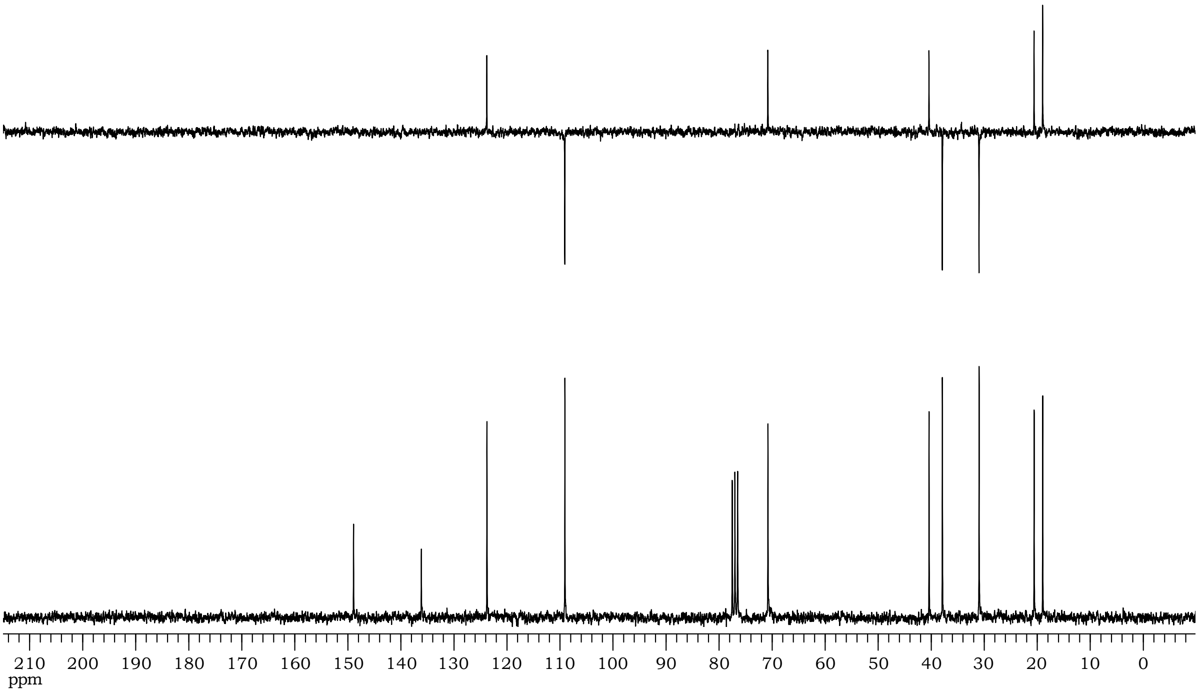

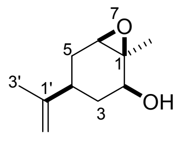

6a

^{1}H-RMN (400 MHz, CDCl$_3$): δ 4.64 (sa, 2H, CH$_2$-2'), 3.80 (dd, 1H, J_1 = 14.4 Hz, J_2 = 9.7 Hz, H-1), 3.11 (d, 1H, J = 4.9 Hz, H-5), 2.41 (sa, 1H, OH), 1.94 (m, 2H, H-4 and H-3), 1.71 (dm, 1H, J = 12.1 Hz, H-2), 1.63 (m, 1H, H-2), 1.62 (s, 3H, CH$_3$-3'), 1.40 (s, 3H, CH$_3$-19), 1.31 (c, 1H, J = 12.1 Hz, H-2).

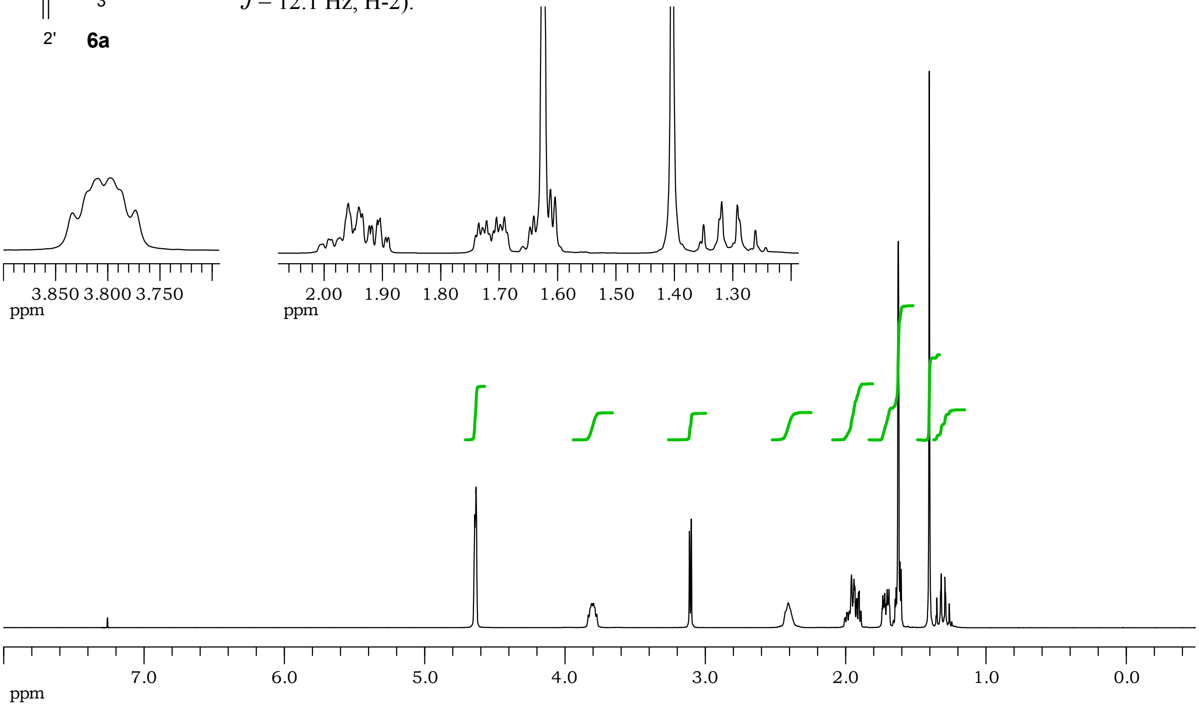

^{13}C-RMN (100.6 MHz, CDCl$_3$): δ 147.5 (C, C-1), 109.6 (CH$_2$, C-2'), 71.9 (CH, C-1), 62.1 (CH, C-5), 60.4 (C, C-10), 40.3 (CH, C-3), 33.7 (CH$_2$, C-2), 29.0 (CH$_2$, C-4), 20.0 (CH$_3$, C-3'), 19.1 (CH$_3$, C-19).

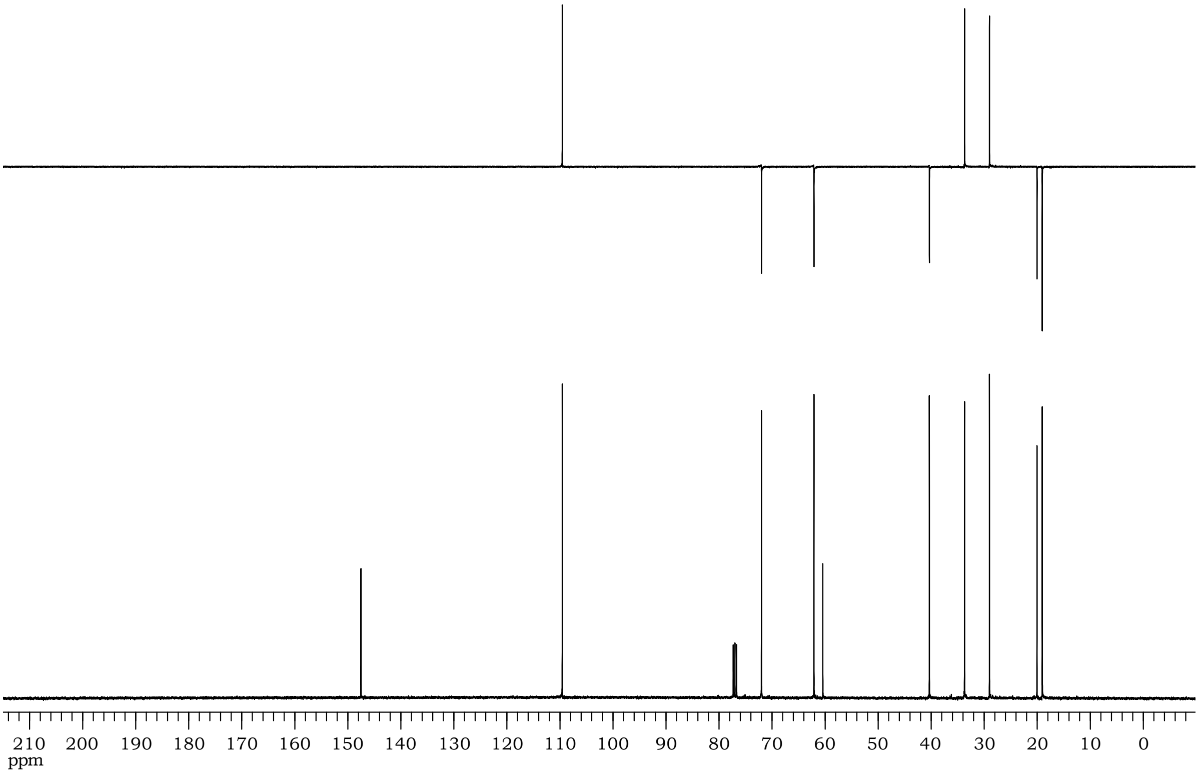

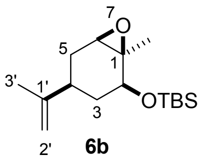

6b

[1]**H-RMN** (250 MHz, $CDCl_3$): δ 4.68 (s, 2H, CH_2-2'), 3.91 (t, 1H, *J* = 7.4 Hz, H-2), 3.04 (d, 1H, *J* = 4.8 Hz, H-6), 1.67 (s, 3H, CH_3-3'), 1.36 (s, 3H, CH_3C-1), 0.91 (s, 9H, Me_3C-Si), 0.10 (s, 3H, Me-Si), 0.08 (s, 3H, Me-Si).

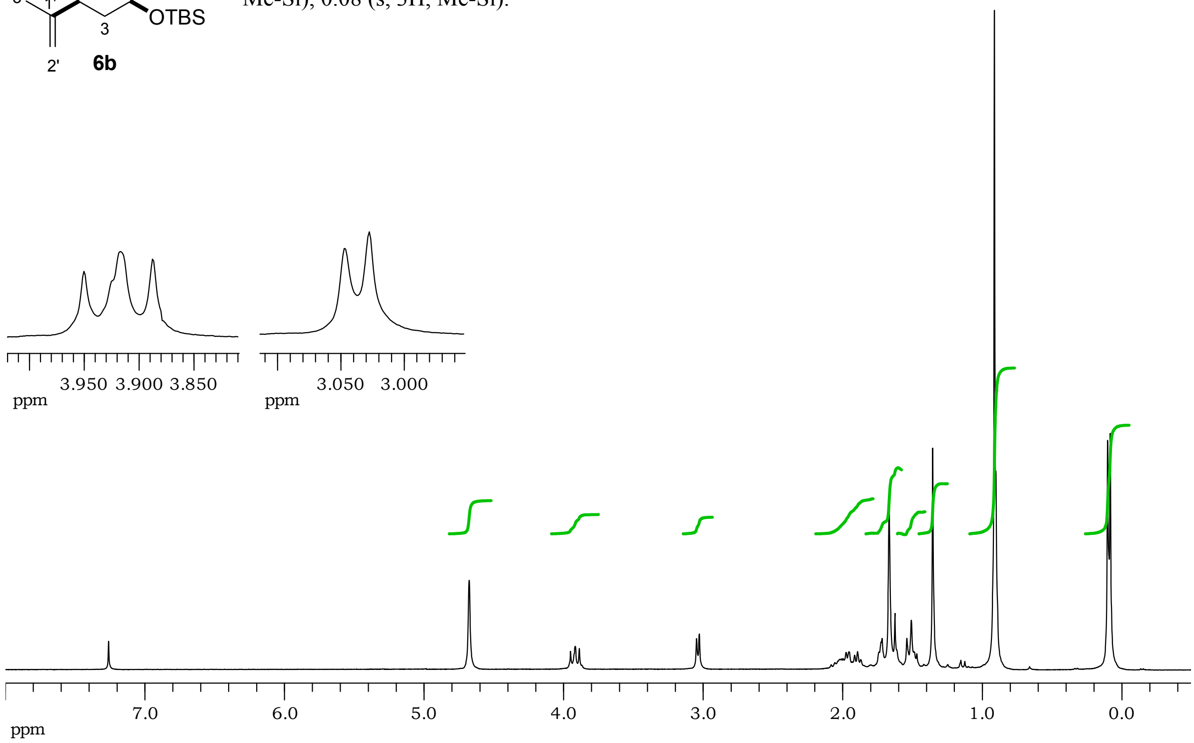

[13]**C-RMN** (62.9 MHz, $CDCl_3$): δ 148.1 (=C, C-1'), 109.6 (CH, C-2'), 73.2 (CH, C-2), 60.9 (CH, C-6), 60.2 (C, C-1), 40.6 (CH, C-4), 33.7 (CH_2, C-3), 29.1 (CH_2, C-5), 25.8 (CH_3, Me_3C-Si), 20.0 (CH_3, C-3'), 19.7 (CH_3, CH_3C-1), 18.1 (C, C-Si), -4.0 (CH_3, Me-Si), -4.7 (CH_3, Me-Si).

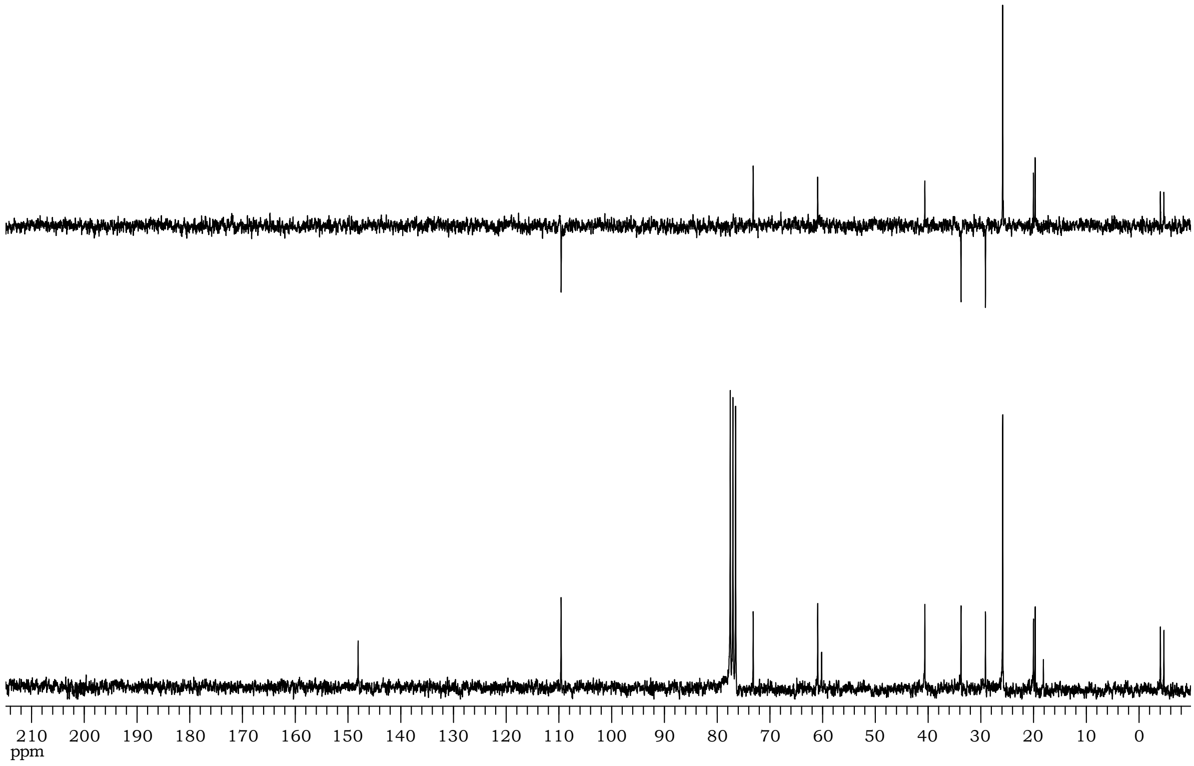

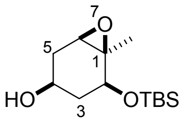

7a

^{1}H-RMN (250 MHz, $CDCl_3$): δ 3.98 (dd, 1H, J_1 = 9.1 Hz, J_2 = 5.5 Hz, H-5), 3.65 (m, 1H, H-3), 2.97 (d, 1H, J = 4.4 Hz, H-1), 2.18 (ddd, 1H, J_1 = 14.8 Hz, J_2 = 6.2 Hz, J_3 = 4.6 Hz, H-2), 2.00 (d, 1H, J =5.5 Hz, OH), 1.80 (dd, 1H, J_1 = 14.8 Hz, J_2 = 8.9 Hz, H-4), 1.74 (1H, m, H-2), 1.63 (dd, 1H, J_1 = 10.6 Hz, J_2 = 9.3 Hz, H-4), 1.34 (s, 3H, CH_3C-6), 0.90 (s, 9H, Me_3C-Si), 0.10 (s, 3H, Me-Si), 0.09 (s, 3H, Me-Si).

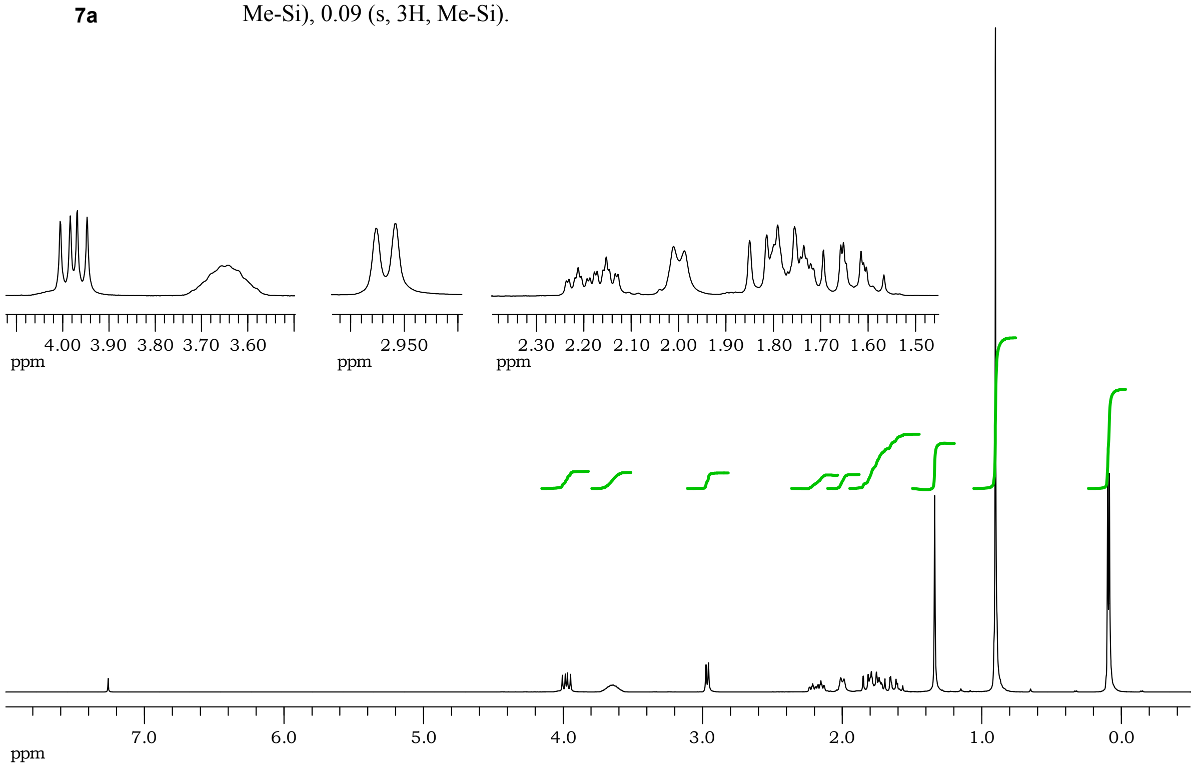

^{13}C-RMN (62.9 MHz, $CDCl_3$): δ 70.6 (CH, C-1), 65.5 (CH, C-3), 60.1 (CH, C-1), 58.6 (C, C-6), 38.0 (CH_2, C-4), 33.2 (CH_2, C-2), 25.8 (3xCH_3, Me_3C-Si), 19.7 (CH_3, CH_3C-6), 18.0 (C, C-Si), -4.2 (CH_3, Me-Si), -4.8 (CH_3, Me-Si).

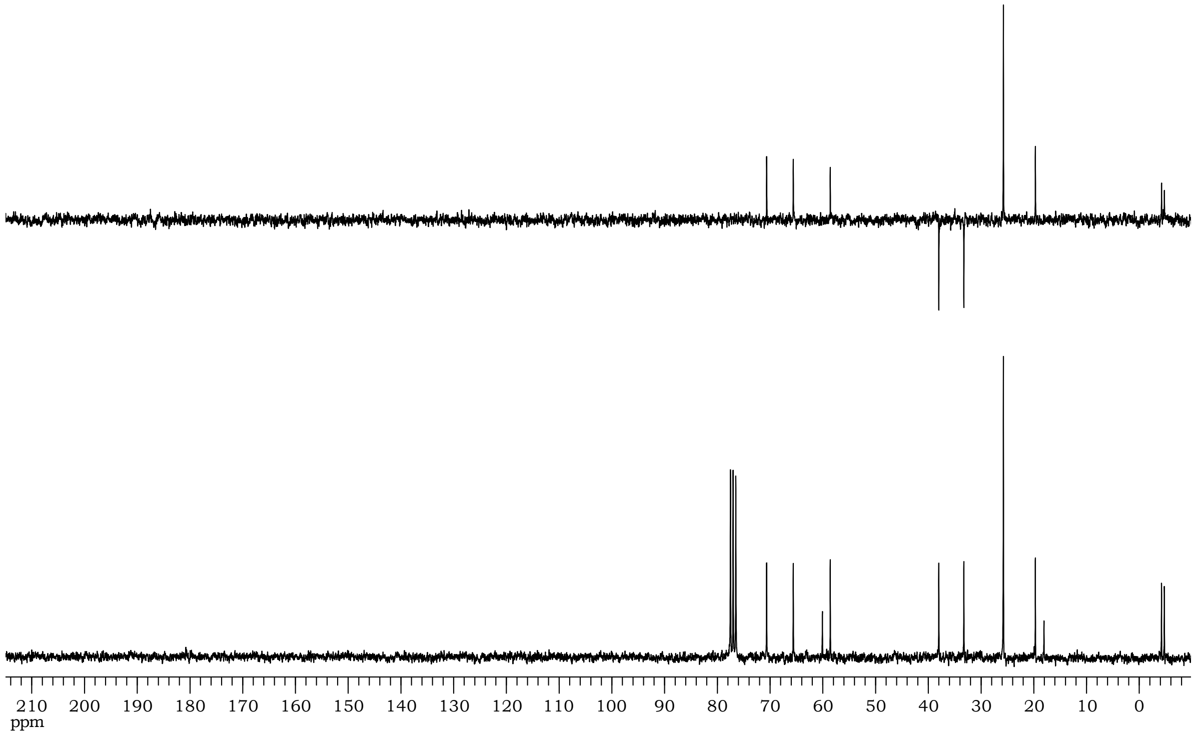

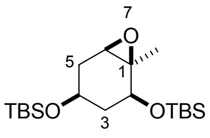

7b

^{1}H-RMN (300 MHz, $CDCl_3$): δ 3.91 (dd, 1H, J_1 = 10.3 Hz, J_2 = 5.6 Hz, H-2), 3.56 (tdd, 1H, J_1 = 11.0 Hz, J_2 = 6.9 Hz, J_3 = 4.4 Hz, H-4), 2.87 (d, 1H, J = 5.1 Hz, H-6), 2.09 (ddd, 1H, J_1 = 14.7 Hz, J_2 = 6.9 Hz, J_3 = 5.2 Hz, H-5), 1.74 (dd, 1H, J_1 = 14.9 Hz, J_2 = 10.0 Hz, H-5), 1.66 (m, 1H, H-3), 1.64 (m, 1H, H-3), 1.32 (s, 3H, CH_3C-1), 0.91 (s, 9H, Me_3C-Si), 0.86 (s, 9H, Me_3C-Si), 0.10 (s, 3H, Me-Si), 0.08 (s, 3H, Me-Si), 0.033 (s, 3H, Me-Si), 0.030 (s, 3H, Me-Si).

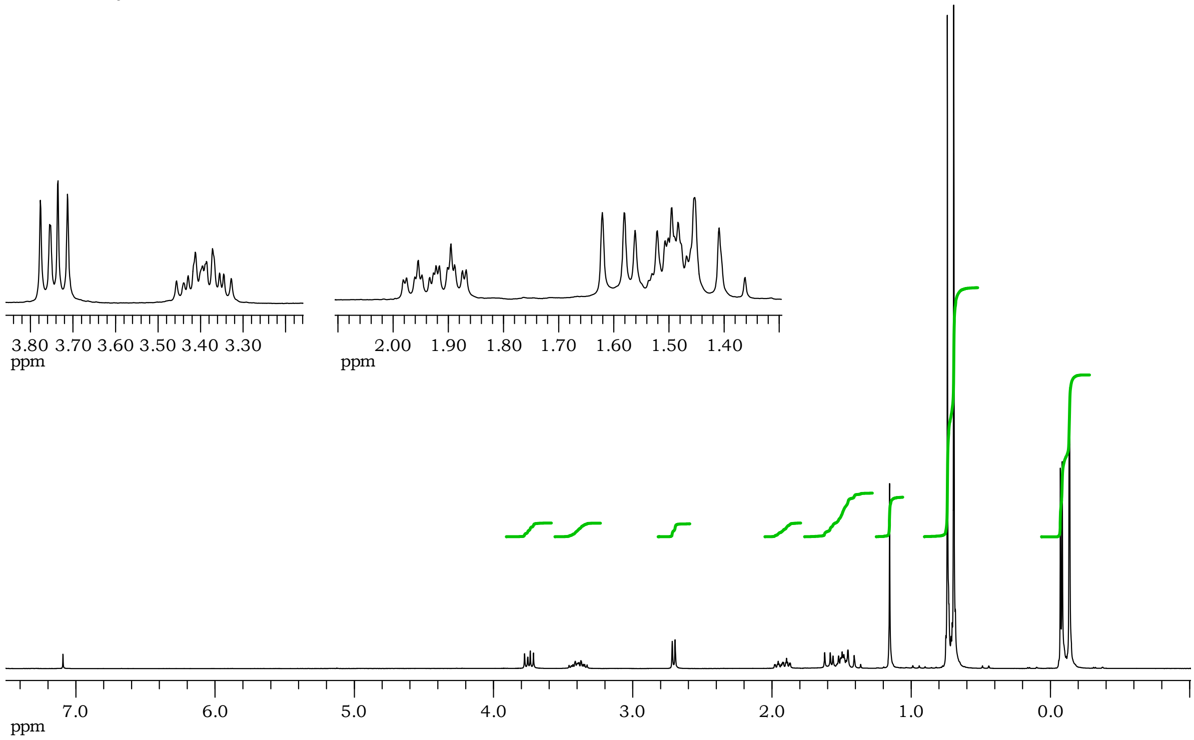

^{13}C-RMN (75 MHz, $CDCl_3$): δ 71.2 (CH, C-2), 66.4 (CH, C-4), 60.1 (C, C-1), 58.2 (CH, C-6), 38.5 (CH_2, C-3), 34.0 (CH_2, C-5), 25.8 (6xCH_3, 2xMe_3C-Si), 19.2 (CH_3, CH_3C-1), 18.1 (2xC, 2xC-Si), -4.2 (CH_3, Me-Si), -4.7 (3xCH_3, Me-Si).

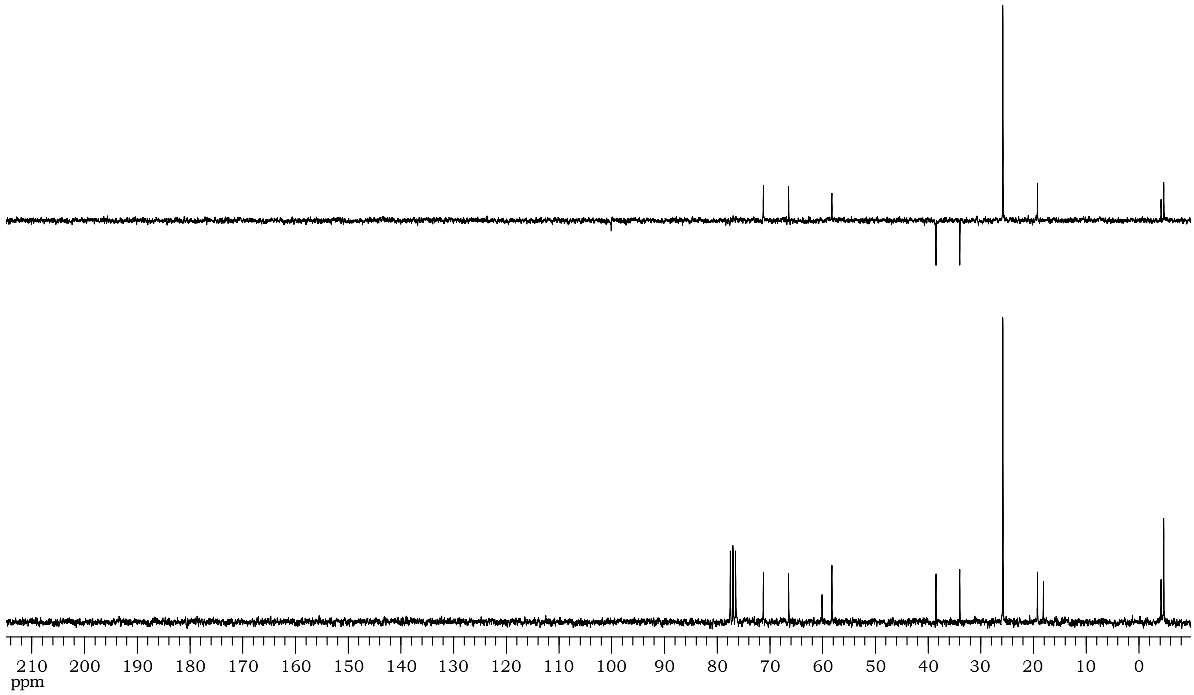

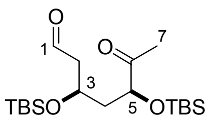

8a

^{1}H-RMN (250 MHz, CDCl$_3$): δ 9.75 (t, 1H, J = 2.2 Hz, H-1), 4.28 (q, 1H, J = 5.9 Hz, H-3), 4.06 (t, 1H, J = 6.4 Hz, H-5), 2.61 (ddd, 1H, J_1 = 16.1 Hz, J_2 = 5.2 Hz, J_3 =1.9 Hz, H-2), 2.51 (ddd, 1H, J_1 = 16.1 Hz, J_2 = 6.6 Hz, J_3 = 2.6 Hz, H-2), 2.13 (s, 3H, C-7), 1.86 (dd, 1H, J_1 = 14.1 Hz, J_2 = 6.6 Hz, H-4), 1.75 (dd, 1H, J_1 = 14.1 Hz, J_2 = 6.4 Hz, H-4), 0.88 (s, 9H, Me$_3$C-Si), 0.83 (s, 9H, Me$_3$C-Si), 0.06 (s, 3H, Me-Si), 0.03 (s, 9H, 3xMe-Si).

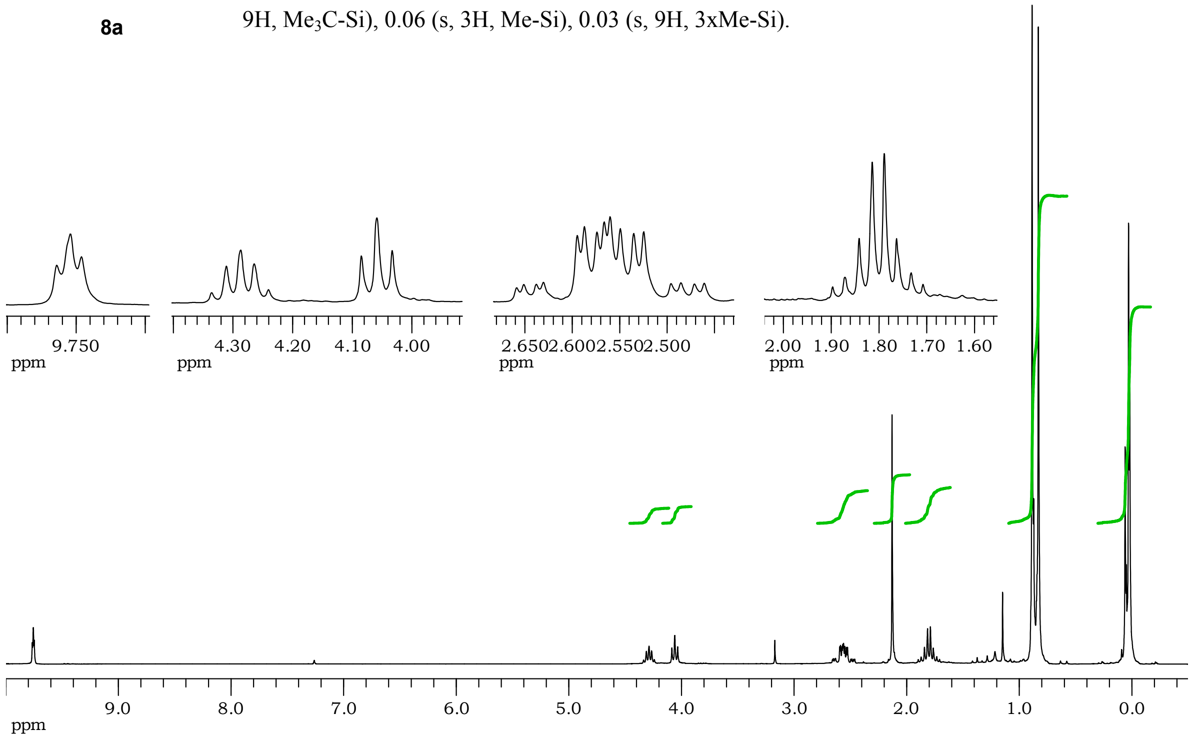

^{13}C-RMN (62.9 MHz, CDCl$_3$): δ 210.7 (C=O, C-6), 201.3 (HC=O, C-1), 75.6 (CH, C-5), 64.7 (CH, C-3), 50.6 (CH$_2$, C-2), 42.1 (CH$_2$, C-4), 25.6 (6xCH$_3$, 2xMe$_3$C-Si), 25.0 (CH$_3$, C-7), 17.9 (2xC, 2xC-Si), -4.7 (CH$_3$, Me-Si), -5.0 (CH$_3$, Me-Si), -5.2 (2xCH$_3$, Me$_2$-Si).

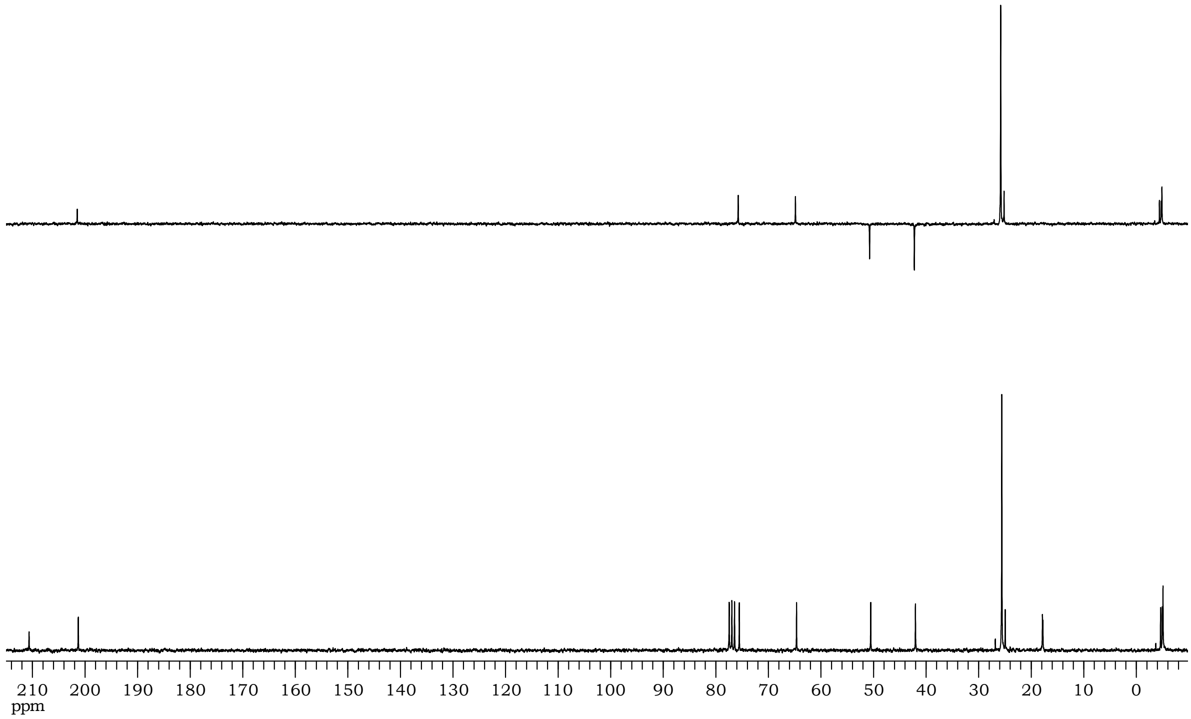

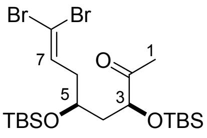

8b

^{1}H-RMN (250 MHz, CDCl$_3$): δ 6.45 (t, 1H, J = 7.2 Hz, H-7), 4.09 (t, 1H, J = 6.5 Hz, H-3), 3.95 (q, 1H, J = 5.7 Hz, H-5), 2.30 (m, 2H, CH$_2$-6), 2.15 (s, 3H, CH$_3$-1), 1.79 (dd, 1H, J_1 =13.9 Hz, J_2 = 6.3 Hz, H-4), 1.69 (dd, 1H, J_1 = 13.9 Hz, J_2 = 6.3 Hz, H-4), 0.92 (s, 9H, Me$_3$C-Si), 0.88 (s, 9H, Me$_3$C-Si), 0.07 (s, 9H, 3xMe-Si), 0.06 (s, 3H, Me-Si).

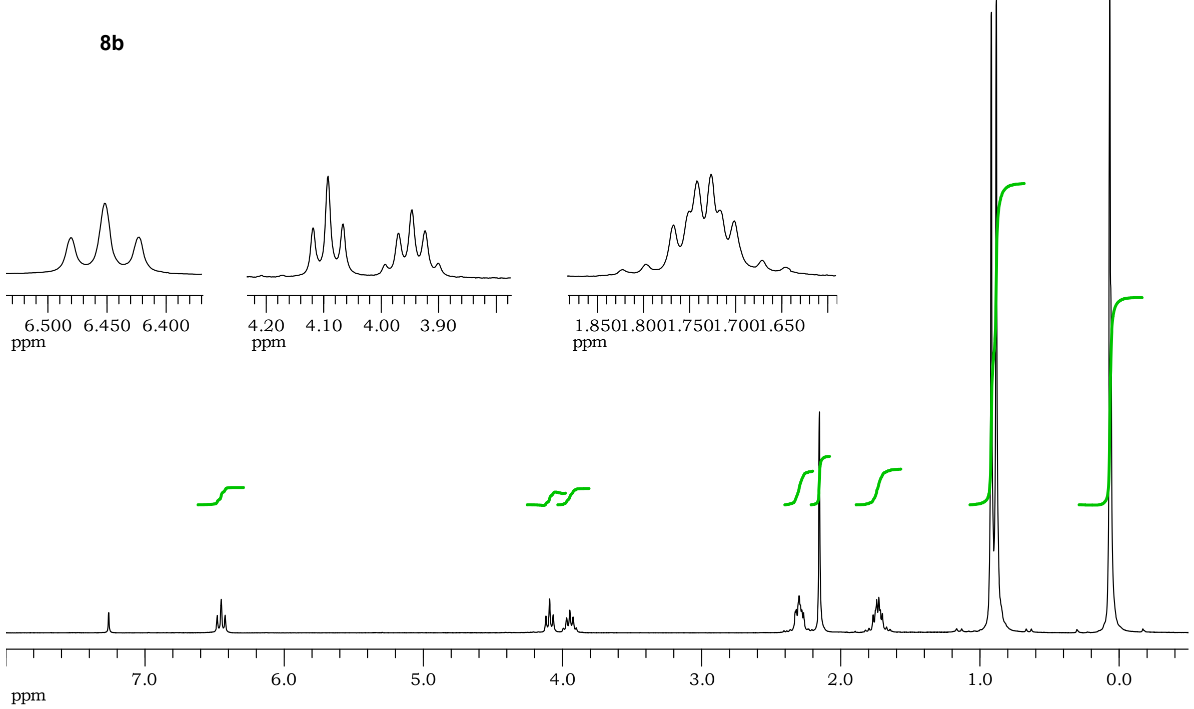

^{13}C-RMN (62.9 MHz, CDCl$_3$): 210.8 (C=O, C-2), 135.0 (=CH, C-7), 90.1 (=C, C-8), 75.7 (CH, C-3), 67.0 (CH, C-5), 41.7 (CH$_2$, C-4), 40.4 (CH$_2$, C-6), 25.8 (3xCH$_3$, Me$_3$C-Si), 25.7 (3xCH$_3$, Me$_3$C-Si), 25.2 (CH$_3$, C-1), 18.0 (C, C-Si), -4.60 (CH$_3$, Me-Si), -4.7 (CH$_3$, Me-Si), -4.86 (CH$_3$, Me-Si), -4.92 (CH$_3$, Me-Si).

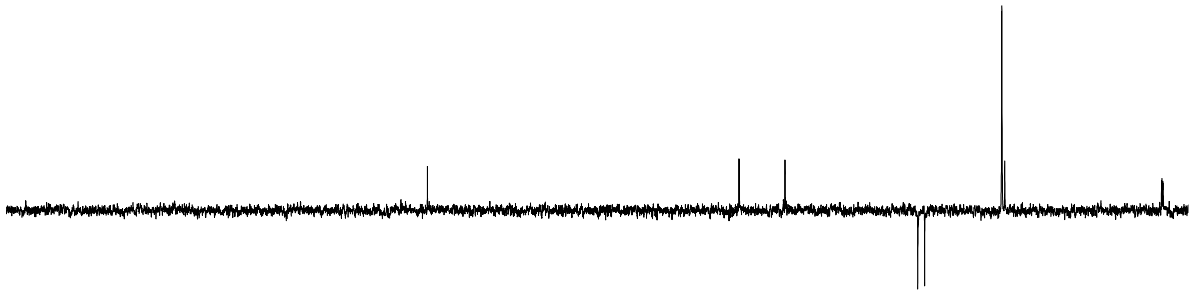

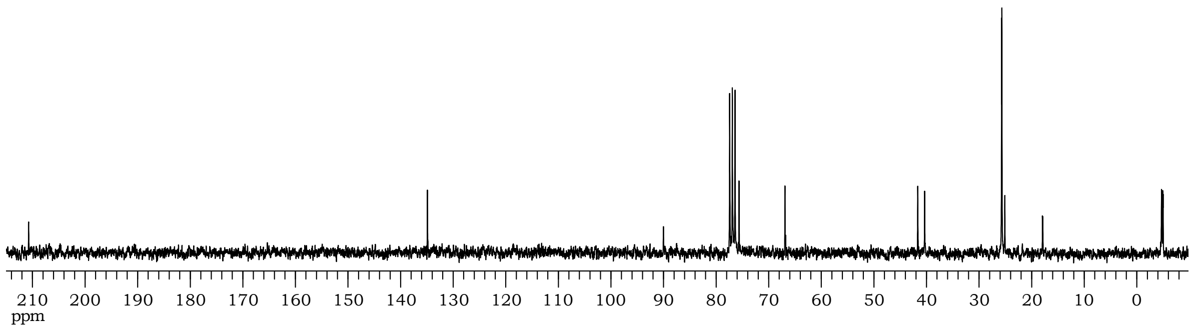

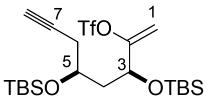

3

^{1}H-RMN (250 MHz, $CDCl_3$): δ 5.21 (d, 1H, J = 3.5 Hz, H-1), 5.19 (d, 1H, J = 3.5 Hz, H-1), 4.39 (t, 1H, J = 6.3 Hz, H-3), 3.86 (q, 1H, J = 5.8 Hz, H-5), 2.38 (m, 2H, CH_2-6), 2.07 (dt, 1H, J_1 = 14.1 Hz, J_2 = 6.0 Hz, H-4), 1.99 (t, 1H, J = 2.6 Hz, H-8), 1.85 (dt, 1H, J_1 = 14.1 Hz, J_2 = 6.3 Hz, H-4), 0.92 (s, 9H, Me_3C-Si), 0.89 (s, 9H, Me_3C-Si), 0.11 (CH_3, Me-Si), 0.09 (CH_3, Me-Si), 0.08 (CH_3, Me-Si), 0.06 (CH_3, Me-Si).

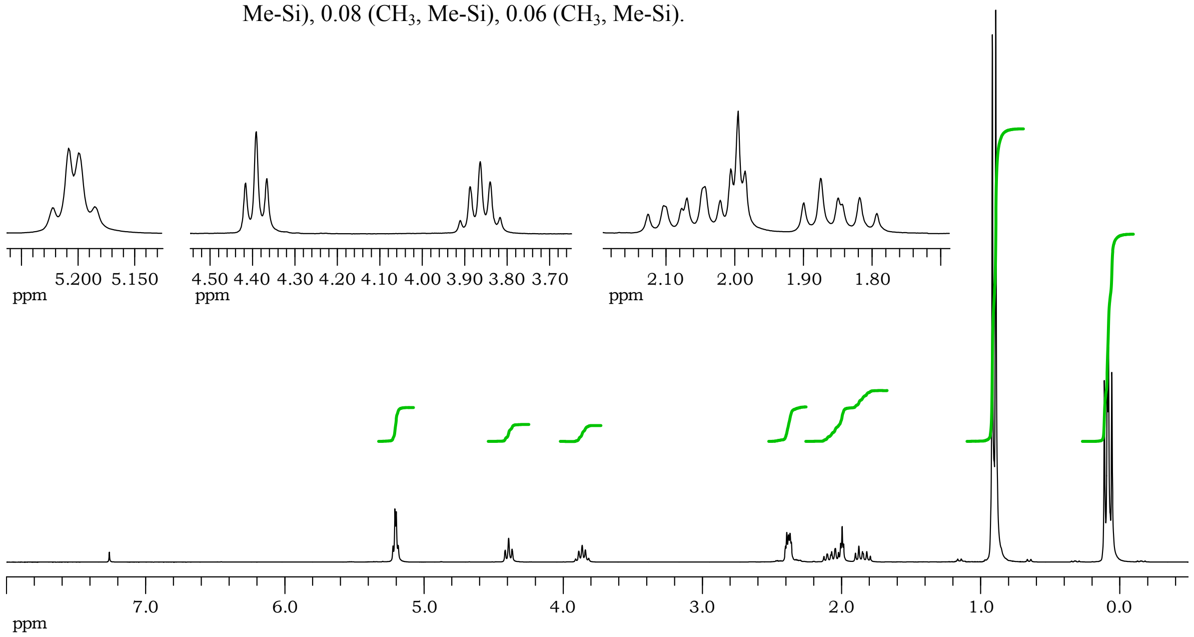

^{13}C-RMN (62.9 MHz, $CDCl_3$): 156.4 (=C, C-2), 104.4 (=CH_2, C-1), 80.7 (≡C, C-7) 70.6 (≡CH, C-8), 69.4 (CH, C-3), 67.4 (CH, C-1), 41.6 (CH_2, C-4), 27.4 (CH_2, C-6), 25.74 (CH_3, Me_3C-Si), 25.70 (CH_3, Me_3C-Si), 18.05 (C, C-Si), 17.99 (C, C-Si), -4.3 (CH_3, Me-Si), -4.8 (CH_3, Me-Si), -4.9 (CH_3, Me-Si), -5.1 (CH_3, Me-Si).

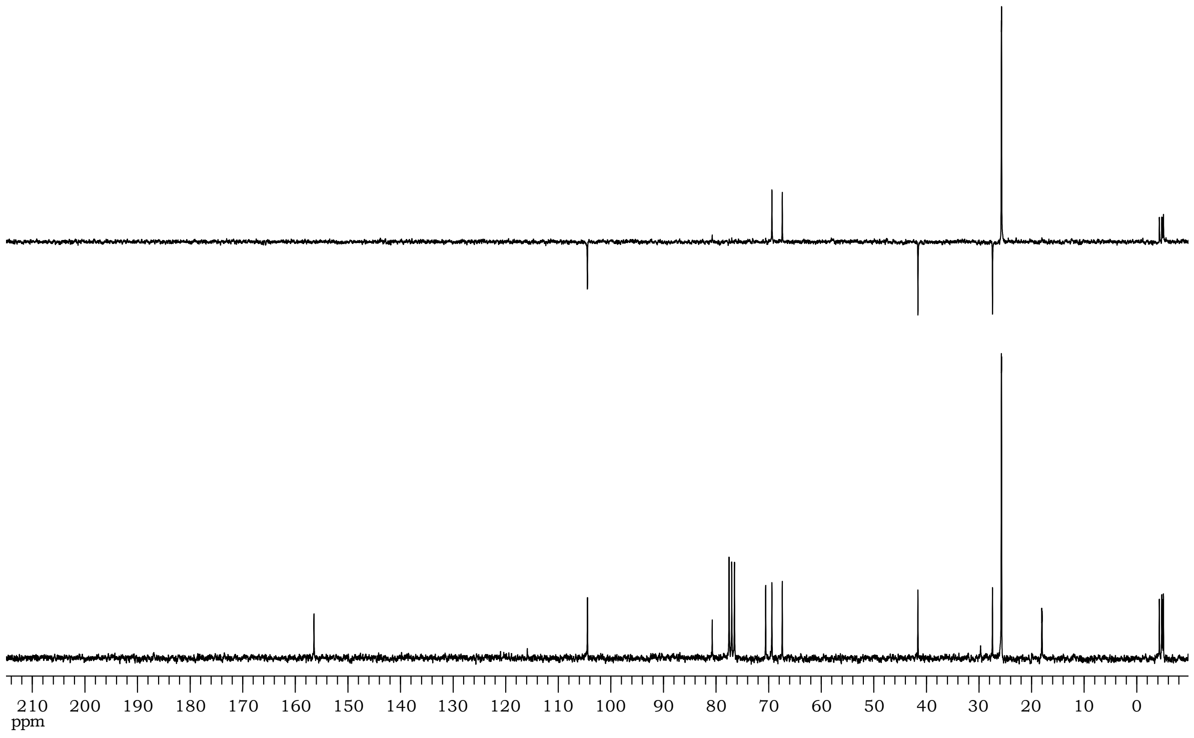

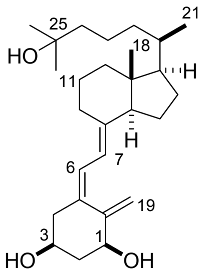

1b

[1]**H-RMN** (500 MHz, $CDCl_3$): δ 6.43 (dd, 1H, J = 11.2 Hz, H-6), 6.02 (dd, 1H, J = 11.3 Hz, H-7), 5.29 (s, 1H, H-19), 5.00 (d, 1H, J = 1.7 Hz, H-19), 4.31 (d, 1H, J = 4.5 Hz, H-1), 4.05 (m, 1H, H-3), 2.84 (dm, 1H, J = 12.5 Hz, H-9), 2.56 (dd, J_1 = 13.5 Hz, J_2 = 2.3 Hz, H-4α), 2.43 (dd, 1H, J_1 = 13.4 Hz, J_2 = 5.7 Hz, H-4β), 2.07 (dt, 1H, J_1 = 13.5 Hz, J_2 = 3.63 Hz, H-12), 1.21 (s, 6H, CH_3-26 and CH_3-27), 0.94 (d, 3H, J = 6.5 Hz, CH_3-21), 0.54 (s, 3H, CH_3-18).

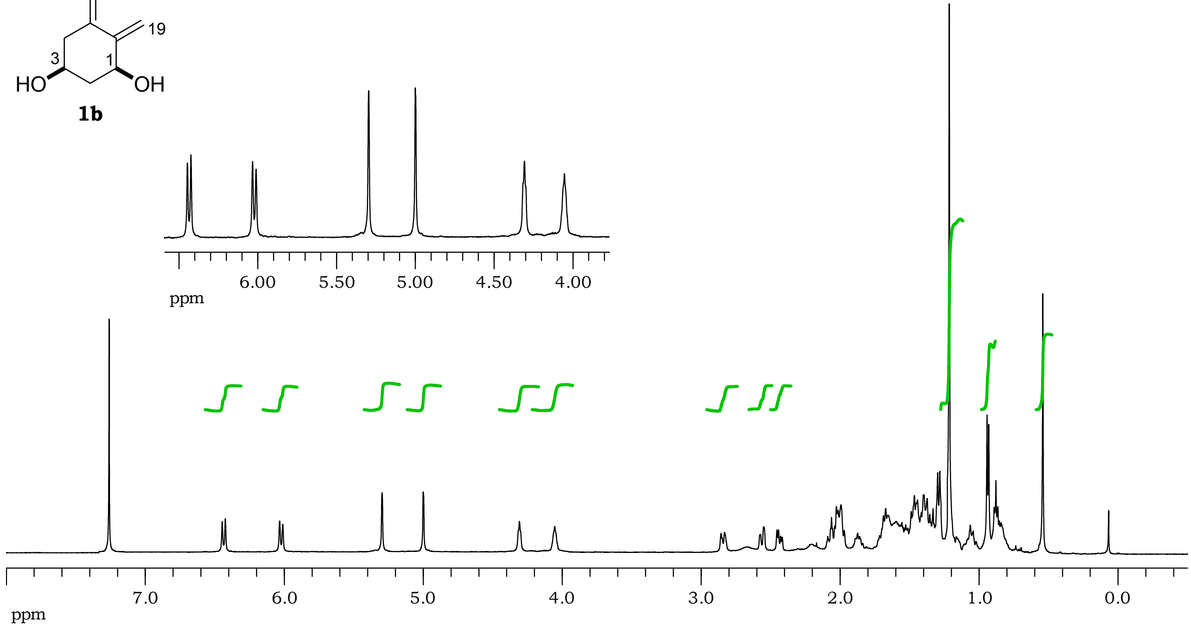

[13]**C-RMN** (125.7 MHz, $CDCl_3$): δ 147.2 (=C, C-10), 143.2 (=C, C-8), 131.6 (=C, C-25), 125.6 (=CH, C-6), 117.0 (=CH, C-7), 112.9 (=CH_2, C-19), 73.1 (CH, C-1), 71.1 (C, C-25), 68.2 (CH, C-3), 56.5 (CH, C-17), 56.3 (CH, C-14), 45.9 (C, C-13), 45.5 (CH, C-2), 44.4 (CH_2, C-4), 40.7 (CH_2), 40.5 (CH_2), 36.4 (CH_2), 36.1 (CH, C-20), 29.4 (CH_3, C-27), 29.2 (CH_3, C-26), 29.1 (CH_2, C-9), 27.7 (CH_2), 23.5 (CH_2), 22.2 (CH_2), 20.8 (CH_2), 18.8 (CH_3, C-21), 12.0 (CH_3, C-18).

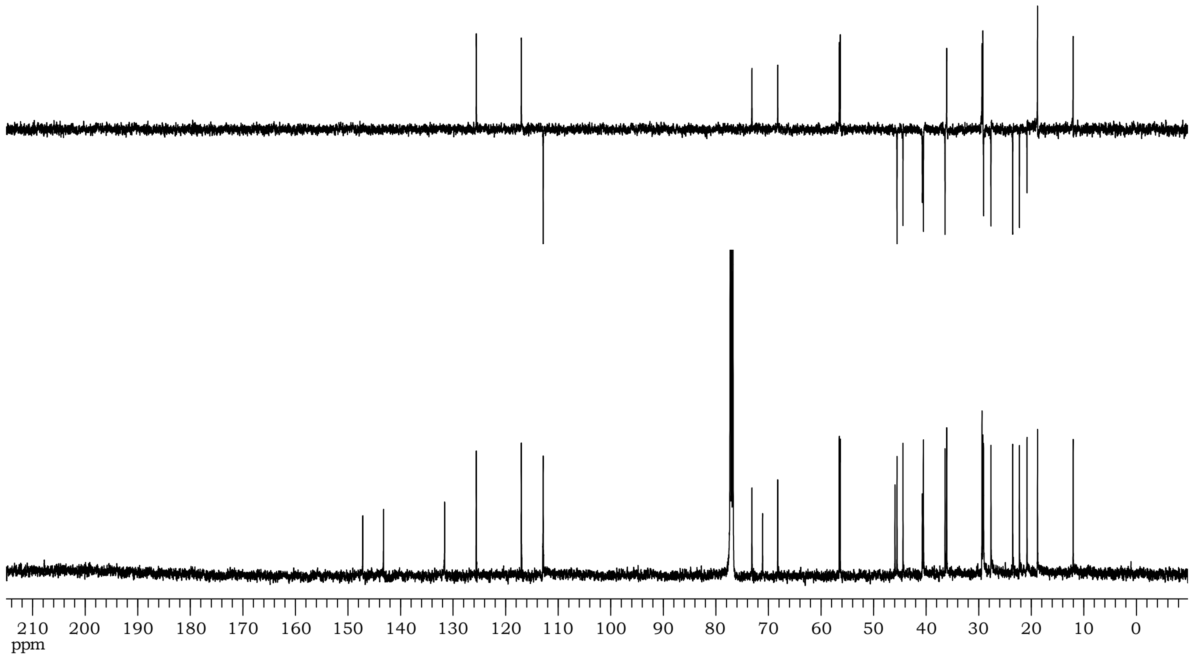

Supplement: Methods S1 — Synthesis. (PDF) [file pone.0018124.s004.pdf]
