# Supplementary material for: 1α,25(OH)2-3-Epi-Vitamin D3, a Natural Physiological Metabolite of Vitamin D3: Its Synthesis, Biological Activity and Crystal Structure with Its Receptor
Source: PLoS One. 2011 Mar 31;6(3):e18124. doi: 10.1371/journal.pone.0018124 (PMC3069065; doi:10.1371/journal.pone.0018124)
Supplement: Methods S2 — (DOCX) [file pone.0018124.s005.docx]

**Methods S2**

***Additional ligands and reference compounds***

Authentic reference compounds for most of the major metabolites detected on keratinocyte incubation, namely 1α,25(OH)2-3-epi-D3, 1α,24,25(OH)3D3, 1α,25(OH)2-24-oxo-D3, 1α,23,25(OH)3D3, 1α,23,25(OH)3-24-oxoD3, 1α,25(OH)2-23,26-lactone D3, were a generous gift of S.Reddy (Brown University) or obtained from Solvay Duphar (Weesp, Netherlands): namely, 24,25(OH)2D3 and 1α,25(OH)2D3). If not otherwise indicated, chemicals and biochemicals of highest purity available were obtained from E.Merck (Darmstadt) and Sigma (St.Louis,Mo).

***HL60 cell culture and differentiation***

HL60 cells were cultured in complete RPMI with 10% fetal calf serum (Sigma) and 5% gentamycine (Kalys) at 37°C without CO2 as previously described (56). Cells were plated at a density of 4x105 cells/ml and cultured for 96 hours in the presence of 1α,25(OH)2D3, or 1α,25(OH)2-3-epi-D3, at various concentrations. Control incubations were performed with 0.7% ethanol. HL60 cell differentiation was determined by flow cytometry using phycoerythrin (PE)-conjugated anti-human CD11c and fluorescein isothiocyanate (FITC)-conjugated antihuman CD14 antibodies (Pharmingen/BD Biosciences). Cellular differentiation was assessed by capillary cytometry (excitation 488nm, emission 520 and 570 nm on the Guava EasyCyte Plus HP system, with absolute cell count and six parameters, Guava Tech. Hayward, CA, USA) and analyzed under Guava Express Pro 5.2.1 (Guava Tech. Hayward, CA, USA).

***HPLC-analysis of incubation of extracts from human keratinocytes***

HPLC-analysis of the CHCl3-extracts was done on a Zorbax-Sil column (Dupont, 4,6x250mm) using a nonlinear gradient from 97:3 to 85:15 hexane: 2-propanol at a flow rate of 2ml/min and a total run time of 100min. We used a fully automatic equipment (Hewlett Packard Series 1050), which enabled the continuous recording, integration and storage of the on-line recordings from the UV-flow-through detector (240nM) and the radioactivity flowthrough detector (Radiomatic Flo- One, Canberra Packard). An excellent separation and quantification of still available substrate 25(OH)D3 and of the individual main metabolites formed from it was achieved (quantification limit 0.05pmoles). Substrate and individual major metabolites were assigned to peaks by matching with unlabeled reference compounds in co-chromatography. (25(OH)D3, 24,25(OH)2D3, 1α,25(OH)2D3, 1α,25(OH)2-3-epi-D3, 1α,24,25(OH)3D3, 1α,25(OH)2-24-oxo D3, 1α,23,25(OH)3D3, 1α,23,25(OH)3-24-oxo D3, 1α,25(OH)2-23,26-lactoneD 3). The concentration of a distinct product was determined from its fraction in the chromatogram corrected for the yield of 3H-activity in the CHCl3-extract applied to the HPLC-column.
