# Supplementary figures and images for: 1α,25(OH)2-3-Epi-Vitamin D3, a Natural Physiological Metabolite of Vitamin D3: Its Synthesis, Biological Activity and Crystal Structure with Its Receptor
Source: PLoS One. 2011 Mar 31;6(3):e18124. doi: 10.1371/journal.pone.0018124 (PMC3069065; doi:10.1371/journal.pone.0018124)

**SCHEME 1: Retrosynthesis of 1b.**

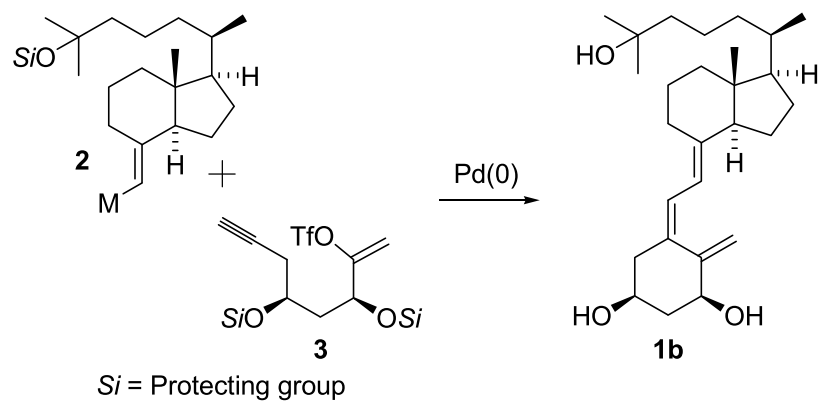

Supplement: Scheme S1 — Retrosynthesis of 1b. (PDF) [file pone.0018124.s006.pdf]

**SCHEME 2: Synthesis of enol triflate 3.**

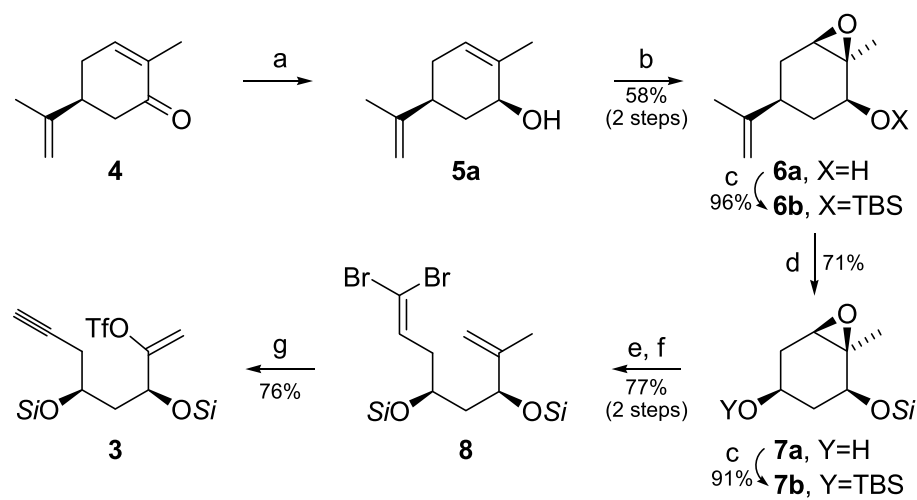

Supplement: Scheme S2 — Synthesis of enol triflate 3. Si = TBS = Si(t-Bu)(CH3)2.TBHP = t-BuOOH (a) NaBH4, CeCl3·7H2O, MeOH, 0°C, 30 min. (b) TBHP, VO(acac)2, PhH, reflux, 30 min. (c) TBSCl, Im, DMF, rt, 12 h. (d) O3, MeOH-CH2Cl2, −78°C; Ac2O, Et3N, DMAP, −35°C to −8°C, 2 h; NaOAc, MeOH, 37°C, 12 h, (e) H5IO6, Et2O, rt, 2 h. (f) CBr4, Zn, Ph3P, CH2Cl2, rt, 40 min. (g) LDA, THF, −78°C, 1 h; n-BuLi, 15 min; 5-Cl-Py-2NTf2, −78°C to rt, 12 h. (PDF) [file pone.0018124.s007.pdf]

**SCHEME 3: Synthesis of metabolite 1.**

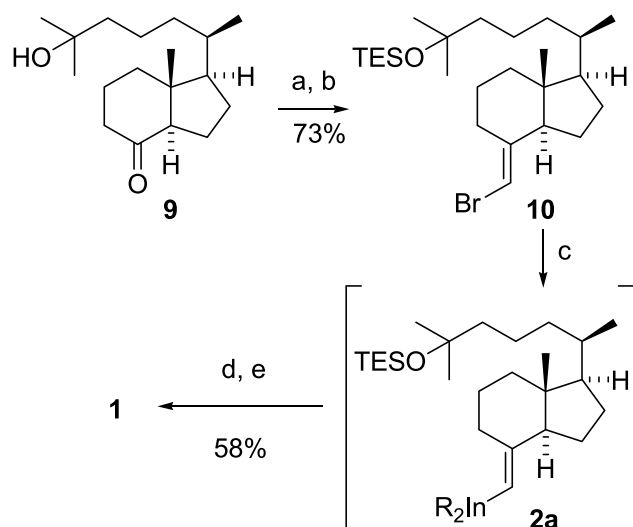

Supplement: Scheme S3 — Synthesis of metabolite 1. TES = Si(CH2CH3)3. (a) (Ph3PCH2Br)Br, KOt-Bu, toluene, −5°C to rt, 1 h, 80%. (b) TESCl, Im, DMAP, DMF, rt, 3 h, 91%. (c) InCl3, t-BuLi, THF, −78°C to 0°C, 2 h. (d) 3, (Ph3P)4Pd, Et3N, THF, (dppf)PdCl2, 0°C to rt, 12 h. (e) HF·Py, Et3N, CH2Cl2, CH3CN, rt, 4 h, 58%. (PDF) [file pone.0018124.s008.pdf]
